# Integrative analysis reveals associations between oral microbiota dysbiosis and host genetic and epigenetic aberrations in oral cavity squamous cell carcinoma

**DOI:** 10.1038/s41522-024-00511-x

**Published:** 2024-04-08

**Authors:** Liuyang Cai, Hengyan Zhu, Qianqian Mou, Po Yee Wong, Linlin Lan, Cherrie W. K. Ng, Pu Lei, Man Kit Cheung, Daijuanru Wang, Eddy W. Y. Wong, Eric H. L. Lau, Zenon W. C. Yeung, Ronald Lai, Katie Meehan, Sherwood Fung, Kwan Chee A. Chan, Vivian W. Y. Lui, Alfred S. L. Cheng, Jun Yu, Paul K. S. Chan, Jason Y. K. Chan, Zigui Chen

**Affiliations:** 1 Department of Microbiology, The Chinese University of Hong Kong, Hong Kong SAR, China; 2 Department of Otorhinolaryngology, Head and Neck Surgery, The Chinese University of Hong Kong, Hong Kong SAR, China; 3 Department of Chemical Pathology, The Chinese University of Hong Kong, Hong Kong SAR, China; 4 Georgia Cancer Center, Augusta, GA 30912 USA; 5 Department of Medicine, Medical College of Georgia, Augusta University, Augusta, GA 30912 USA; 6 School of Biomedical Sciences, The Chinese University of Hong Kong, Hong Kong SAR, China; 7 Department of Medicine and Therapeutics, The Chinese University of Hong Kong, Hong Kong SAR, China

**Keywords:** Clinical microbiology, Microbiota, Clinical microbiology

## Abstract

Dysbiosis of the human oral microbiota has been reported to be associated with oral cavity squamous cell carcinoma (OSCC) while the host-microbiota interactions with respect to the potential impact of pathogenic bacteria on host genomic and epigenomic abnormalities remain poorly studied. In this study, the mucosal bacterial community, host genome-wide transcriptome and DNA CpG methylation were simultaneously profiled in tumors and their adjacent normal tissues of OSCC patients. Significant enrichment in the relative abundance of seven bacteria species (*Fusobacterium nucleatum*, *Treponema medium*, *Peptostreptococcus stomatis*, *Gemella morbillorum*, *Catonella morbi*, *Peptoanaerobacter yurli* and *Peptococcus simiae*) were observed in OSCC tumor microenvironment. These tumor-enriched bacteria formed 254 positive correlations with 206 up-regulated host genes, mainly involving signaling pathways related to cell adhesion, migration and proliferation. Integrative analysis of bacteria-transcriptome and bacteria-methylation correlations identified at least 20 dysregulated host genes with inverted CpG methylation in their promoter regions associated with enrichment of bacterial pathogens, implying a potential of pathogenic bacteria to regulate gene expression, in part, through epigenetic alterations. An in vitro model further confirmed that *Fusobacterium nucleatum* might contribute to cellular invasion via crosstalk with E-cadherin/β-catenin signaling, TNFα/NF-κB pathway and extracellular matrix remodeling by up-regulating *SNAI2* gene, a key transcription factor of epithelial-mesenchymal transition (EMT). Our work using multi-omics approaches explored complex host-microbiota interactions and provided important insights into genetic and functional basis in OSCC tumorigenesis, which may serve as a precursor for hypothesis-driven study to better understand the causational relationship of pathogenic bacteria in this deadly cancer.

## Introduction

Oral cavity squamous cell carcinoma (OSCC) is the most prevalent oral malignancy worldwide and is associated with significant mortality and morbidity rates^1^. About 60% of oral cancers are diagnosed at an advanced stage, resulting in a 5-year survival rates of less than 50%. Known etiological factors of OSCC tumorigenesis include tobacco consumption, alcohol abuse and betel nut chewing; however, the prevalence of OSCC without traditional risk factors has been increasing in recent years^2^. There is therefore an urgent need to identify other underlying etiologies with prognostic relevance for early diagnosis and improved treatment in OSCC patients. Given the increasing evidence indicating the carcinogenic potential of the human microbiome in cancers^3^, elucidating the human microbiome in OSCC may explain, in part, the fact that a subset of patients who are not exposed to traditional risk factors eventually develop cancer. In fact, a significant loss in microbial diversity has been reported in OSCC patients^4^; several periodontal pathogens, for example, *Fusobacterium*, *Peptostreptococcus* and *Treponema*, were significantly enriched in OSCC tumor microenvironment^5,6^. These pathogenic bacteria were reported to promote oral cancer aggressivity via TLR/MyD88 triggered activation of integrin/FAK signaling pathway^7^, making the understanding of pathogenic bacteria in cancer of great importance.

There is a growing awareness that global patterns of genetic and epigenetic changes play a critical role in the molecular characteristics of oral cancer. For example, *EGFR*, *PIK3CA*, *NOTCH* pathways and *TP53* gene are among the most frequently altered in head and neck squamous cell carcinoma (HNSCC)^8,9^. Several studies of head and neck cancer have identified promoter methylation of *CDKN2A (p16)*, DAP kinase (*DAPK*), and DNA repair genes *MGMT* and *MLH1*^10,11^. Bacteria could trigger human epigenetic modification, thereby silencing tumor suppressor gene expression^12^. Thus, profiling DNA genetic and epigenetic events that might be affected by dysbiosis of the oral microbiota may provide us with a key understanding of its role in gene regulation and enable us in establishing specific marker for early diagnosis and therapeutic strategies.

In this study, OSCC tumors and their adjacent normal (AN) tissues from a cohort of HPV-negative patients were simultaneously profiled for oral mucosal microbiota, host genome-wide transcriptome and DNA CpG methylation to explore the genetic basis of host-microbiota interactions. Integrative analysis using multi-omics approaches revealed complex networks between oral microbiota dysbiosis and host genetic and epigenetic abnormalities. Our findings provide important insights into genetic and functional basis for better understanding the role of oral bacteria in the pathogenesis of OSCC.

## Results

### Study subjects

A total of 98 OSCC patients who provided paired tumor and AN tissues were recruited in this study. Among them, eight patients infected with high-risk HPV types (7 with HPV16 and one with HPV18) were excluded. This retained cohort consisted of 57 males and 33 females, with a mean age of 65 years (sd: 12 years). Detailed demographic and clinical information are available in Supplementary Tables 1 and 2.

### Oral microbiota dysbiosis in OSCC

**Fig. 1:**
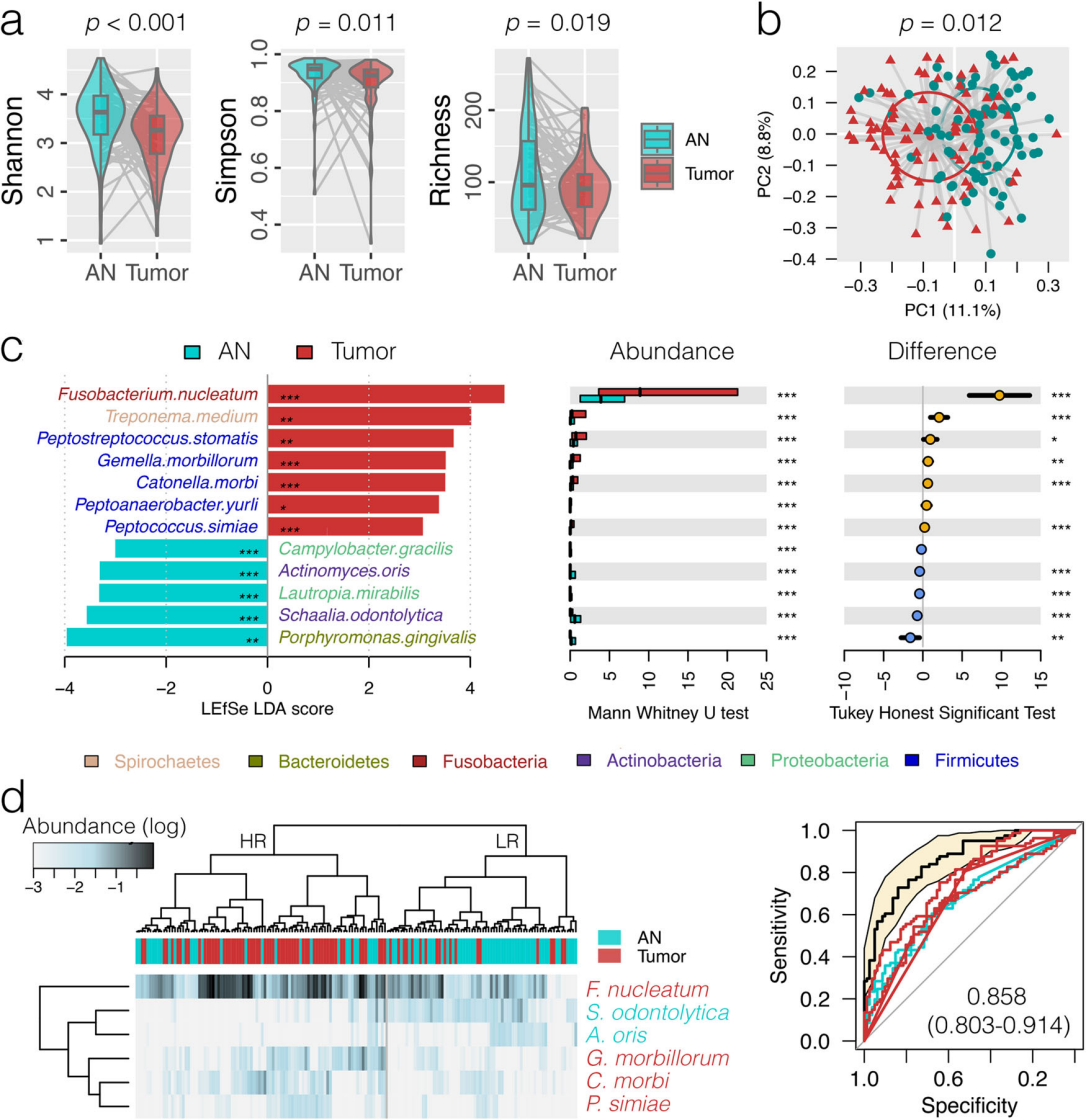
Oral microbiota dysbiosis associated with oral cavity squamous cell carcinoma (OSCC). **a** Comparison of the oral microbiota alpha diversity between OSCC tumor (tumor) and adjacent normal (AN) tissues at the amplicon sequence variant (ASV) level. The boxplot’s center line indicates the median value, the box bounds represent the first and third quartiles, and the whiskers extend to the smallest and largest values in the data, respectively. **b** Principal coordinate analysis plot based on weighted GUniFrac distance matrix inferred from ASVs. **c** Discriminative bacterial species as detected by linear discriminant analysis (LDA) effect size (LEfSe) analysis (score > 3, *q* < 0.05), which was further validated by at least two of the three compositional aware tools, ANCOM-BC2, ALDEx2 and ZicoSeq tests adjusted for the covariates of T stage and smoking (*q* < 0.05). The bar length represents log10 LDA score. Differences in the relative abundance were further tested by pairwise Mann–Whitney *U* test and Tukey HSD post hoc as shown on the right panels. *p < 0.05; **p < 0.01; ***p < 0.001. **d** Hierarchical cluster analysis using distance matrix of six discriminative bacterial species (LDA > 3, AUC > 0.65) classified the surveyed tissue samples into two clades.

High quality bacterial 16S reads from paired tumor and AN tissues were available from 81 OSCC patients (mean reads of 32,698 ± 20,799) (Supplementary Table 3 and Supplementary Data 1–3), with Firmicutes (mean relative abundance of 31.2 ± standard deviation of 1.3%) as the most predominant bacterial phylum, followed by Fusobacteria (21.2 ± 1.3%), Proteobacteria (20.5 ± 1.5%), Bacteroidetes (16.8 ± 0.7%) and seven other phyla (Supplementary Fig. 1a). At the amplicon sequence variant (ASV) level, significantly reduced alpha diversity of the oral mucosal microbial community was observed in tumor tissues as measured by Richness, Shannon and Simpson indices compared to AN controls (Mann–Whitney *U* test, *p* < 0.019) (Fig. 1a); similarly, a principal coordinate analysis (PCoA) based on the weighted GUniFrac matrix showed distinct separation between tumor and AN groups (*p* = 0.012) (Fig. 1b). The finding underscores the distinct microbial community associated with OSCC tumor sites compared to adjacent normal tissues.

We used permutational multivariate analysis of variance (PERMANOVA) to quantify the contribution of factors (disease status, gender, aging, smoking, alcohol consumption, and T and N cancer stages) to the differences of microbial composition in patients with OSCC. This analysis identified disease status as the independent factor that had the largest effect on the overall structure of the oral microbiota (weighted GUniFrac *R*^2^ = 0.0411, *p* < 0.001; unweighted GUniFrac *R*^2^ = 0.0234, *p* < 0.001; Bray–Curtis *R*^2^ = 0.0221, *p* < 0.001) (Supplementary Fig. 1b). In addition, other factors, including T stage (T1&T2 vs. T3&T4) (weighted GUniFrac *R*^2^ = 0.0172, *p* < 0.001; unweighted GUniFrac *R*^2^ = 0.0169, *p* < 0.001; Bray–Curtis *R*^2^ = 0.0132, *p* < 0.001) and smoking (Yes vs. No) (weighted GUniFrac *R*^2^ = 0.0104, *p* = 0.013; Bray–Curtis *R*^2^ = 0.0097, *p* = 0.002) were also significant, albeit with a smaller effect. The influence of disease status on oral microbiota composition highlights its potential role in OSCC pathogenesis.

Using a linear discriminant analysis effect size (LEfSe) test (LDA > 3, *q* < 0.05), which was further verified by at least two of the three compositional aware tools, ANCOM-BC2, ALDEx2 and ZicoSeq tests adjusted for the covariates of T stage and smoking (*q* < 0.05), we applied a phylogenetic placement algorithm, pplacer, to map ASV reads against a NCBI 16S rRNA reference database with maximum likelihood and observed 6 and 13 bacterial genera significantly enriched and depressed in the relative abundance in OSCC tumor microenvironment, respectively (Supplementary Fig. 1c, Table 1 and Supplementary Table 4a). Moreover, seven mucosal bacterial species (*Fusobacterium nucleatum*, *Treponema medium*, *Peptostreptococcus stomatis*, *Gemella morbillorum*, *Catonella morbi*, *Peptoanaerobacter yurli* and *Peptococcus simiae*) were found to be predominantly colonized in the oral cavity of cancer patients (Fig. 1c, Table 1 and Supplementary Table 4b), suggesting a potential of tumor-enriched bacteria in the pathogenesis of OSCC.

**Table 1:** Discriminative bacterial genus and species observed in OSCC tumor tissues compared with the paired adjacent normal tissues

	Mean abundance (±sd) (%)		LEfSe test			Tukey HSD test		MWU test		ANCOM-BC2 test^a^				ALDEx2 test^a^			ZicoSeq test^a^		Representative ASV
Taxa	AN	Tumor	LDA group	LDA score	LDA *q* value	Difference (%)	*q* value	*p* value	*q* value	logFC	*w* value	*p* value	*q* value	*t* value	*p* value	*q* value	*p* value	*q* value	
Genus																			
*Fusobacterium*	8.75 ± 0.76	21.20 ± 1.74	Tumor	**4.78**	**0.0000**	12.45 (8.71, 16.20)	**0.0000**	0.0000	**0.0000**	1.12	4.27	0.0000	**0.0002**	5.72	0.0000	**0.0001**	0.0600	**0.0979**	
*Treponema*	1.16 ± 0.23	5.24 ± 1.10	Tumor	**4.29**	**0.0180**	4.07 (1.85, 6.29)	**0.0004**	0.0001	**0.0028**	0.81	2.57	0.0114	**0.0235**	2.57	0.0156	0.9819	0.0100	**0.0017**	
*Peptoanaerobacter*	0.17 ± 0.04	1.37 ± 0.36	Tumor	**3.82**	**0.0017**	1.19 (0.47, 1.92)	**0.0014**	0.0000	**0.0001**	1.46	6.16	0.0000	**0.0000**	3.58	0.0012	0.3104	0.0100	**0.0070**	
*Peptostreptococcus*	0.91 ± 0.18	2.11 ± 0.44	Tumor	**3.80**	**0.0019**	1.20 (0.26, 2.14)	**0.0125**	0.0004	**0.0066**	0.71	2.74	0.0070	**0.0151**	3.77	0.0004	0.1432	0.0300	**0.0468**	
*Catonella*	0.27 ± 0.06	0.88 ± 0.16	Tumor	**3.54**	**0.0001**	0.62 (0.29, 0.95)	**0.0003**	0.0000	**0.0003**	0.72	3.23	0.0017	**0.0044**	3.94	0.0003	0.1222	0.0100	**0.0063**	
*Peptococcus*	0.12 ± 0.03	0.35 ± 0.06	Tumor	**3.06**	**0.0002**	0.23 (0.10, 0.36)	**0.0008**	0.0001	**0.0018**	0.87	4.23	0.0001	**0.0003**	3.78	0.0008	0.2074	0.0100	**0.0017**	
*Streptococcus*	14.04 ± 1.26	6.65 ± 1.07	AN	**4.55**	**0.0000**	−7.39 (−10.66, −4.13)	**0.0000**	0.0000	**0.0000**	−0.91	−3.64	0.0004	**0.0013**	−3.04	0.0046	0.7240	0.0100	**0.0313**	
*Veillonella*	5.37 ± 0.59	1.40 ± 0.24	AN	**4.30**	**0.0000**	−3.97 (−5.22, −2.71)	**0.0000**	0.0000	**0.0000**	−1.11	−3.95	0.0001	**0.0005**	−3.98	0.0002	0.0660	0.0100	**0.0067**	
*Rothia*	3.38 ± 0.61	1.58 ± 0.68	AN	**3.98**	**0.0000**	−1.80 (−3.62, 0.01)	0.0518	0.0000	**0.0000**	−1.27	−4.40	0.0000	**0.0001**	−4.38	0.0001	**0.0220**	0.0100	**0.0208**	
*Granulicatella*	1.74 ± 0.23	0.79 ± 0.21	AN	**3.69**	**0.0000**	−0.96 (−1.58, −0.34)	**0.0027**	0.0000	**0.0000**	−0.85	−3.21	0.0017	**0.0044**	−4.02	0.0002	0.0768	0.0100	**0.0428**	
*Schaalia*	1.28 ± 0.17	0.42 ± 0.12	AN	**3.64**	**0.0000**	−0.85 (−1.26, −0.45)	**0.0000**	0.0000	**0.0000**	−0.98	−4.03	0.0001	**0.0004**	−4.00	0.0003	0.0867	0.0100	**0.0013**	
*Actinomyces*	0.77 ± 0.12	0.09 ± 0.02	AN	**3.57**	**0.0000**	−0.68 (−0.93, −0.43)	**0.0000**	0.0000	**0.0000**	−1.08	−5.32	0.0000	**0.0000**	−4.92	0.0000	**0.0073**	0.0100	**0.0081**	
*Lautropia*	0.49 ± 0.13	0.08 ± 0.05	AN	**3.36**	**0.0002**	−0.42 (−0.68, −0.15)	**0.0023**	0.0000	**0.0005**	−0.87	−4.08	0.0002	**0.0007**	−3.18	0.0059	0.5965	0.0100	**0.0013**	
*Corynebacterium*	0.46 ± 0.11	0.10 ± 0.04	AN	**3.30**	**0.0000**	−0.36 (−0.59, −0.14)	**0.0018**	0.0000	**0.0002**	−0.75	−3.53	0.0008	**0.0026**	−3.96	0.0006	0.1612	0.0100	**0.0013**	
*Eubacterium*	0.69 ± 0.13	0.31 ± 0.07	AN	**3.28**	**0.0393**	−0.38 (−0.67, −0.09)	**0.0117**	0.0007	**0.0113**	−0.56	−2.42	0.0174	**0.0337**	−0.63	0.5459	1.0000	0.0100	**0.0208**	
*Halomonas*	0.29 ± 0.19	0.00 ± 0.00	AN	**3.22**	**0.0024**	−0.28 (−0.65, 0.09)	0.1305	0.0045	0.0545	−1.15	−6.82	0.0000	**0.0001**	−1.99	0.1134	0.9503	0.0100	**0.0470**	
*Megasphaera*	0.39 ± 0.09	0.12 ± 0.04	AN	**3.16**	**0.0028**	−0.27 (−0.47, −0.08)	**0.0063**	0.0003	**0.0060**	−0.63	−2.98	0.0042	**0.0096**	−2.42	0.0295	0.9625	0.0100	**0.0072**	
*Lancefieldella*	0.62 ± 0.10	0.39 ± 0.12	AN	**3.11**	**0.0115**	−0.24 (−0.55, 0.08)	0.1447	0.0001	**0.0018**	−1.06	−4.66	0.0000	**0.0001**	−1.20	0.2663	1.0000	0.0100	**0.0070**	
*Phocaeicola*	0.26 ± 0.09	0.04 ± 0.02	AN	**3.04**	**0.0040**	−0.22 (−0.41, −0.03)	**0.0212**	0.0006	**0.0109**	−0.74	−3.57	0.0008	**0.0027**	−2.49	0.0387	0.8908	0.0100	**0.0070**	
Species																			
*Fusobacterium nucleatum*	5.54 ± 0.67	15.29 ± 1.83	Tumor	**4.68**	**0.0000**	9.75 (5.91, 13.59)	**0.0000**	0.0000	**0.0000**	1.15	4.54	0.0000	**0.0001**	3.92	0.0002	0.1623	0.0100	**0.0229**	4c305539242bc00f72118f6a70d5d103
*Treponema medium*	0.39 ± 0.07	2.46 ± 0.55	Tumor	**4.02**	**0.0030**	2.06 (0.97, 3.16)	**0.0003**	0.0000	**0.0013**	1.01	4.01	0.0001	**0.0005**	3.15	0.0036	0.8050	0.0100	**0.0043**	88d5e4fcfd68b0cef9f267bd04d6b934
*Peptostreptococcus stomatis*	0.81 ± 0.18	1.76 ± 0.40	Tumor	**3.67**	**0.0021**	0.95 (0.08, 1.82)	**0.0323**	0.0005	**0.0094**	0.67	2.78	0.0062	**0.0132**	3.79	0.0004	0.2569	0.0100	**0.0183**	feb281c58c97b847d8e32aa9dae2174b
*Gemella morbillorum*	0.39 ± 0.10	1.06 ± 0.20	Tumor	**3.52**	**0.0000**	0.67 (0.23, 1.11)	**0.0034**	0.0000	**0.0011**	0.39	1.93	0.0569	0.0879	4.73	0.0000	**0.0140**	0.0100	**0.0488**	66358cc06be2067caaeab6047c327466
*Catonella morbi*	0.27 ± 0.06	0.88 ± 0.16	Tumor	**3.51**	**0.0001**	0.62 (0.29, 0.95)	**0.0003**	0.0000	**0.0005**	0.71	3.45	0.0008	**0.0024**	4.00	0.0003	0.1628	0.0100	**0.0092**	de1e79ff05735a3eb21b59e27188410c
*Peptoanaerobacter yurli*	0.08 ± 0.04	0.56 ± 0.24	Tumor	**3.38**	**0.0145**	0.47 (0.00, 0.95)	0.0514	0.0008	**0.0133**	1.08	5.64	0.0000	**0.0000**	2.61	0.0246	0.9239	0.0100	**0.0213**	74a1666eb102cd17c22028f87467e2e4
*Peptococcus simiae*	0.12 ± 0.03	0.35 ± 0.06	Tumor	**3.06**	**0.0002**	0.23 (0.10, 0.36)	**0.0008**	0.0001	**0.0019**	0.88	4.68	0.0000	**0.0001**	3.76	0.0011	0.3176	0.0100	**0.0025**	e5944afd1dc39fe0c43f907049fd6ac4
*Porphyromonas gingivalis*	1.88 ± 0.58	0.28 ± 0.13	AN	**3.96**	**0.0016**	−1.60 (−2.77, −0.42)	**0.0080**	0.0000	**0.0003**	−0.78	−3.33	0.0013	**0.0037**	−2.62	0.0179	0.9642	0.0100	**0.0051**	204ae45c366a21148f2675b55295831c
*Schaalia odontolytica*	1.08 ± 0.16	0.37 ± 0.11	AN	**3.57**	**0.0000**	−0.72 (−1.11, −0.32)	**0.0004**	0.0000	**0.0000**	−0.78	−3.63	0.0004	**0.0015**	−3.86	0.0004	0.2285	0.0200	**0.0043**	845954a9086d53d7993b092f560b4fcc
*Lautropia mirabilis*	0.46 ± 0.12	0.03 ± 0.01	AN	**3.32**	**0.0004**	−0.42 (−0.66, −0.19)	**0.0005**	0.0000	**0.0007**	−0.93	−5.00	0.0000	**0.0001**	−2.86	0.0166	0.8482	0.0100	**0.0025**	14e61b2c1523a6a67df3d576d3c01fdc
*Actinomyces oris*	0.44 ± 0.08	0.03 ± 0.01	AN	**3.31**	**0.0000**	−0.41 (−0.57, −0.25)	**0.0000**	0.0000	**0.0000**	−0.86	−5.31	0.0000	**0.0000**	−4.51	0.0001	0.0855	0.0100	**0.0025**	37e783541d8270c9329ce720d8331512
*Campylobacter gracilis*	0.20 ± 0.10	0.02 ± 0.01	AN	**3.00**	**0.0009**	−0.18 (−0.37, 0.01)	0.0679	0.0000	**0.0013**	−0.72	−4.73	0.0000	**0.0001**	–2.50	0.0324	0.9517	0.0100	**0.0124**	42cb1a145d5871ce13065a619775cab6

Bold values indicate comparisons with statistical significance.

^a^Adjusted for the covariates of T stage and smoking.

A hierarchical clustering inferred from 6 discriminative bacterial species (LDA > 3, AUC > 0.65) was able to differentiate the surveyed samples into two clades, composed of the majority of tumors (62/92) and controls (51/70), with an odds ratio of 5.48 (95% CI 2.66–11.68, *p* < 0.001) (Fig. 1d). These bacterial species achieved a combined area under the receiver operating characteristic (ROC) curve (AUC) of 0.858 (95% CI 0.803–0.914). This discrimination may serve as a basis for developing diagnostic markers for OSCC.

### *Fusobacterium nucleatum* associated with OSCC patients without traditional risk factors

Since *F. nucleatum* was the most predominant bacterial species in OSCC tumor microenvironment (15.29% vs. 5.54%) (Supplementary Fig. 2a and Table 1), we further analyzed its colonization associated with patient features. The enrichment of *F. nucleatum* was significantly associated with non-smokers (mean log2-fold change of 1.48 vs. 0.13, *p* = 0.004) (Supplementary Fig. 2b) and non-drinkers (1.35 vs. 0.27, *p* = 0.034) (Supplementary Fig. 2c). Since all recruited patients were high-risk HPV-negative, the finding implies the potential of pathogenetic role of *F. nucleatum* in OSCC patients negative for HPV infection, smoking and alcohol consumption (triple negative). *F. nucleatum* was also associated with better cancer-specific survival (1.42 vs. 0.27, *p* = 0.042) (Supplementary Fig. 2d), which was likely supported by a better 3-year disease-specific survival (81.0% vs. 58.0% at 36 months, *p* = 0.079) (Supplementary Fig. 2e). These results suggest that *F. nucleatum* could be a distinctive marker in HPV-negative OSCC cases, particularly among non-smokers and non-drinkers, and may also be indicative of a more favorable prognosis in these patients.

### Differentially expressed host gene transcriptome in OSCC

**Fig. 2:**
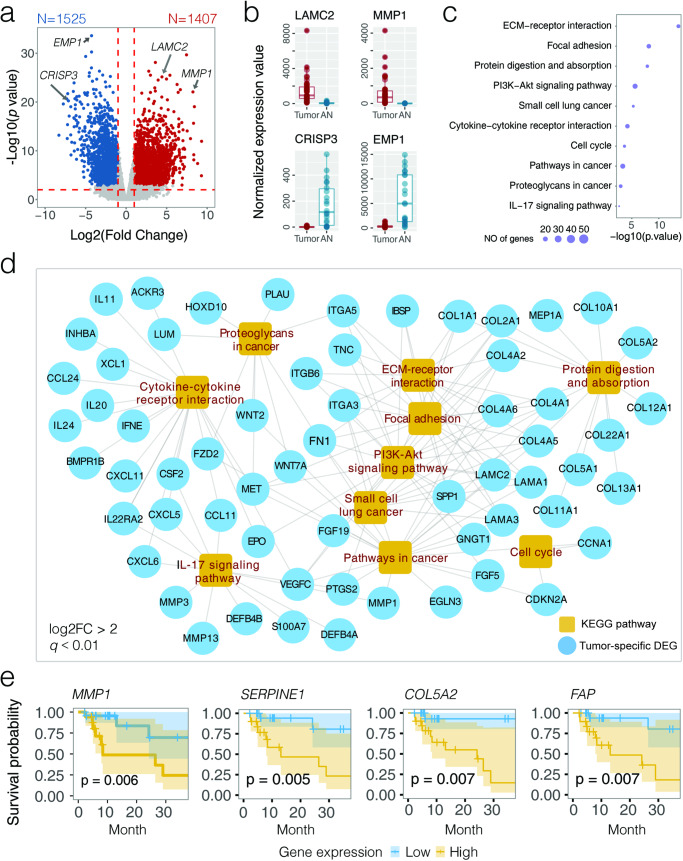
Host transcriptome profiling in OSCC by lncRNA-seq. **a** Volcano plot showing differentially expressed genes (DEGs; abs(log2FC) > 1, *q* < 0.01) between OSCC tumor (*N* = 40) and AN (*N* = 22) tissues. Dots in red and blue indicate up- and down-regulated DEGs in tumors when compared to AN tissues, respectively. **b** Expression of four representative genes (*LAMC2*, *MMP1*, *EMP1*, and *CRISP2*) involved in OSCC tumorigenesis. **c** Top 10 Kyoto Encyclopedia of Genes and Genomes (KEGG) pathways significantly enriched by up-regulated DEGs in OSCC (*q* < 0.05). Sizes of the circles indicate the number of DEGs in the pathway. **d** KEGG gene network visualizing up-regulated DEGs in OSCC (log2FC > 2, *q* < 0.01). **e** Kaplan–Meier estimate for 3-year disease-specific survival based on the abundance levels of four DEGs (*MMP1*, *SERPINE1*, *COL5A2*, and *FAP*). The log-rank test was used to determine significance.

A subset of 40 tumors and 22 AN tissues were profiled for host genome-wide transcriptome (Supplementary Tables 2 and 5), which revealed a globally reconfigured host gene dysregulation in OSCC tumor microenvironment (Supplementary Fig. 3a and Supplementary Table 6). Among 17,225 transcripts with mean transcripts per kilobase million greater than 1 (TPM > 1), we identified 1407 up-regulated (tumor-associated) and 1525 down-regulated (AN-associated) genes in the surveyed OSCC cohort (log2FC > 1 or <−1, *q* < 0.01) (Supplementary Figs. 3a and 3b and Supplementary Table 6), highly consistent with the transcriptome profile of The Cancer Genome Atlas (TCGA) OSCC data (Spearman cor = 0.70, *p* < 0.001) (Supplementary Fig. 3c). For example, the expression of *LAMC2* (involved in epithelial cell migration) was dramatically elevated in both HK-OSCC (log2FC = 4.42, *q* = 1.31E−23) (Fig. 2b) and TCGA-OSCC datasets (Supplementary Fig. 3d). Likewise, *MMP1* (involved in tumor cell invasion) was ranked as one of the most up-regulated genes (log2FC = 8.16, *q* = 9.05E−19). Examples of down-regulated genes included tumor suppressors *CRISP3* (log2FC = −7.15, *q* = 3.36E−17) and *EMP1* (log2FC = −4.34, *q* = 4.21E−29).

An enrichment analysis categorizing 1292 annotated tumor-associated differentially expressed genes (DEGs) (log2FC > 1, *q* < 0.01) into the Kyoto Encyclopedia of Genes and Genomes (KEGG) database found that most altered canonical pathways were related to carcinogenic signaling and immune regulation (*q* < 0.05) (Fig. 2c and Supplementary Table 7). For example, extracellular matrix–receptor (ECM-receptor) interaction (*p* = 2.35E−14) involving the regulation of cellular movement, proliferation and differentiation was the most enriched pathway, in which a variety of DEGs that encode collagen, integrin and laminin were highly expressed (log2FC > 2, *q* < 0.01) (Fig. 2d). Disorders of a variety of metabolism pathways were observed when the annotated down-regulated DEGs (*n* = 1417) were summarized for functional analysis (Supplementary Fig. 3e and Supplementary Table 7). For instance, the downregulation of tyrosine metabolism has been implicated in tumor progression of several types of cancers such as esophageal cancer^13^, oral cancer^14^, and hepatocellular carcinoma^15^.

Survival analysis further identified 62 tumor- and 48 AN-associated DEGs with prognostic values for OSCC (*p* < 0.01) (Supplementary Table 8). These included *MMP1* (involved in proliferation and differentiation) (*p* = 0.006), *SERPINE1* (involved in tumor migration and tissue remodeling) (*p* = 0.005), *COL5A2* (involved in cellular proliferation and invasion) (*p* = 0.007), and *FAP* (involved in familial adenomatous polyposis) (*p* = 0.007) that were significantly associated with poor disease-specific survival (Fig. 2e). The identification of these DEGs provides a prognostic landscape that could be instrumental for risk stratification and personalized treatment planning in OSCC.

### Interactions between oral microbiota and host transcriptome in OSCC

**Fig. 3:**
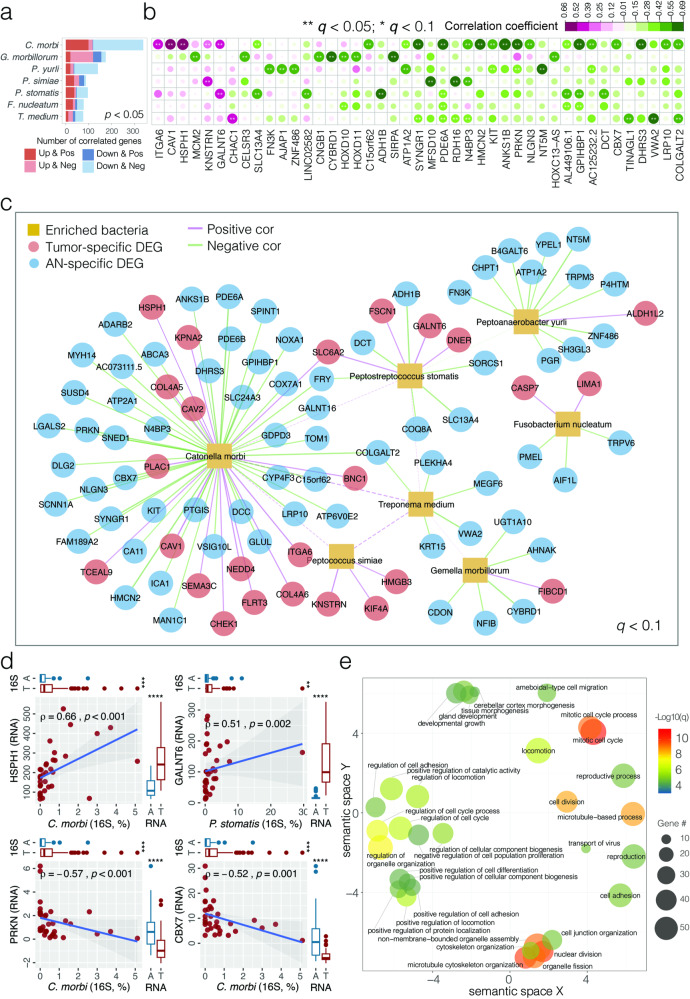
Interactions between tumor-enriched bacterial species and host DEGs associated with OSCC. **a** Bar plot of bacteria-transcriptome Spearman correlation showing the abundance of host DEGs in OSCC tumor tissues associated with tumor-enriched bacterial species (*p* < 0.05). Up and Down indicate up-regulated and down-regulated DEGs, respectively; Pos and Neg indicate positive and negative associations, respectively. *C. morbi* *Catonella morbi*, *G. morbillorum Gemella morbillorum*, *P. yurli* *Peptoanaerobacter yurli*, *P. simiae* *Peptococcus simiae*, *P. stomatis Peptostreptococcus stomatis*, *F. nucleatum Fusobacterium nucleatum*, *T. medium Treponema medium*. **b** Heat map depicting highly significant bacteria-transcriptome correlations (*q* < 0.05). Color panel indicates the Spearman correlation coefficients. Asterisks indicate significance level (**q < 0.05; *q < 0.1). Columns are ranked by the average correlation coefficients of DEGs with tumor-enriched bacteria. **c** Network visualizing significant correlations between bacteria-transcriptome (solid line, Spearman *q* < 0.1) and bacteria-bacteria (dashed line, SparCC correlation *q* < 0.05). **d** Scatter plots showing four representative correlations between bacteria and DEG in OSCC tumor tissues, where the strength of correlation (Spearman rho) and significance (*p* value) are shown in each plot. Marginal boxplots depict overall gene expression (right) and bacterial 16S rRNA gene abundance (top), with Mann–Whitney *U* test between tumor and AN tissues. **e** Gene Ontology (GO) pathways significantly enriched by up-regulat**e**d host DEGs associated with tumor-enriched bacteria. GO terms with functional similarity are clustered in the semantic space by REVIGO. B & H corrected *p* values are indicated by color panel. The size of the circles indicates the number of DEGs associated with the pathways.

In our study, we aim to explore the potential associations between pathogenic bacteria and the transcriptome in OSCC by investigating the correlations between differentially expressed genes (DEGs) and the relative abundance of tumor-enriched bacterial species in OSCC tumor microenvironment. Using Spearman correlations analysis, we noted associations between 814 host DEGs (370 tumor- and 444 AN-associated) and 7 tumor-enriched bacterial species that were frequently found in 34 tumor tissues with available bacterial 16S and host lncRNA-seq data, resulting in 990 statistically significant bacteria-transcriptome associations (*p* < 0.05) (Fig. 3a and Supplementary Table 9a). Notably, among the most significant of these associations (*q* < 0.10), 70.9% (112/158) displayed coherent patterns; that is, a positive correlation of tumor-enriched bacteria with upregulated genes (Up & Positive) or a negative correlation with downregulated genes (Down & Negative) (Fig. 3b, c). For example, we observed a significant positive correlation between the abundance of *C. morbi* and the expression of *HSPH1* (involved in upregulation of Wnt signaling pathway related to cellular proliferation and migration) (rho = 0.660, *p* < 0.001) (Fig. 3d). Similarly, a positive correlation was found between *P. stomatis* abundance and *GALNT6* expression (involved in tumor progression and metastasis) (rho = 0.511, *p* = 0.001). Conversely, tumor suppressor genes such as *PRKN* (rho = −0.571, *p* < 0.001) and *CBX7* (rho = −0.525, *p* = 0.001) exhibited negative correlation with *C. morbi* abundance. Additionally, *COLGALT2*, a gene downregulated in breast cancer, showed a negative correlation with multiple bacterial species including *C. morbi* (rho = −0.528, *p* = 0.001) and *T. medium* (rho = −0.447, *p* = 0.008). It was also noteworthy to mention the positive correlation observed between *C. morbi* and *T. medium* in the surveyed OSCC tumors (SparCC correlation = 0.429, *p* = 0.001) (Fig. 3c and Supplementary Table 9b). We would like to clarify that the identification of these correlations may contribute to a better understanding of the complex interactions between the microbiota and the host response, but they require subsequent functional studies to elucidate any potential causal relationships. Due to the inherent limitations in taxonomic resolution at the species level with the V3–V4 region of the 16 S rRNA gene, we also conducted correlation analyses at the genus level (Supplementary Table 10a, b). These analyses serve as a basis for more targeted hypotheses that could inform future experimental work designed to explore the intricacies of host-microbiota interactions in OSCC.

Up-regulated host DEGs positively associated with tumor-enriched bacterial species (Up & Pos, *N* = 206, *p* < 0.05) were summarized for Gene Ontology (GO) biological processes to characterize the potential of pathogenic bacteria in OSCC pathogenesis (Supplementary Table 11a). As shown in Fig. 3e, the top enriched GO terms included cell cycle, motility, adhesion, proliferation and migration. This was consistent with the Gene Ontology (GO) enrichment results obtained at the genus level (Supplementary Table 11b). For example, *CDK6* was positively associated with *T. medium*; this gene was able to phosphorylate retinoblastoma (Rb) in the G1 phase of the cell cycle by derepressing E2F to promote cellular proliferation. Similarly, several genes encoding proteins involved in tumor shedding, adhesion and migration, such as collagens (*COL4A5*, *COL4A6*), integrins (*ITGB4*) and laminins (*LAMA3*), were overexpressed and positively associated with pathogenic bacteria in OSCC tumor microenvironment.

### Transcriptome deregulation by DNA methylation

Paired tumor and AN tissues from 38 randomly selected OSCC patients were profiled for DNA CpG methylation (Supplementary Tables 2 and 12). A total of 4,584,317 CpG sites with ≥10 reads per sample were characterized, 57.27% of which were located within the promoter regions (Supplementary Fig. 4a). We quantified methylation signals on each chromosome using a 1 kb sliding window to smooth the distribution, which clearly differentiated the surveyed tumors from AN tissues by principal component analysis (PCA, *p* < 0.01) (Supplementary Fig. 4b). We focused on differentially methylated regions (DMRs) mapped to gene promoter regions (up to 3 kb) because of their association with gene expression. Overall, 17,056 hyper- (increased CpG methylation, meth.diff > 1.0) and 26,474 hypo-methylated (decreased CpG methylation, meth.diff < −1.0) promoter regions that regulate 9052 and 13,087 genes, respectively, were identified when comparing OSCC tumors with AN tissues (*q* < 0.01) (Supplementary Fig. 4c and Supplementary Table 13). Hyper-methylated genes included previously reported targets of recurrent hyper-methylation in OSCC, such as *DDAH2*, *CCNA1*, *DCC*, as well as some cancer-associated genes including *HRAS* (Supplementary Fig. 4d). Similarly, examples of hypo-methylated genes included *PI3*, *AIM2*, *PTHLH*, *IFNG* and *CEACAM1*. We designated 477 suppressed and 636 overexpressed DEGs with CpG hyper- (Hyper-Down) and hypo-methylation (Hypo-Up) in their promoter regions, respectively, given the potential of host transcriptome dysregulation by epigenetic modifications (Supplementary Fig. 4e, f and Supplementary Table 14).

### Bacteria-associated epigenetic aberrance on host gene dysregulation in OSCC

**Fig. 4:**
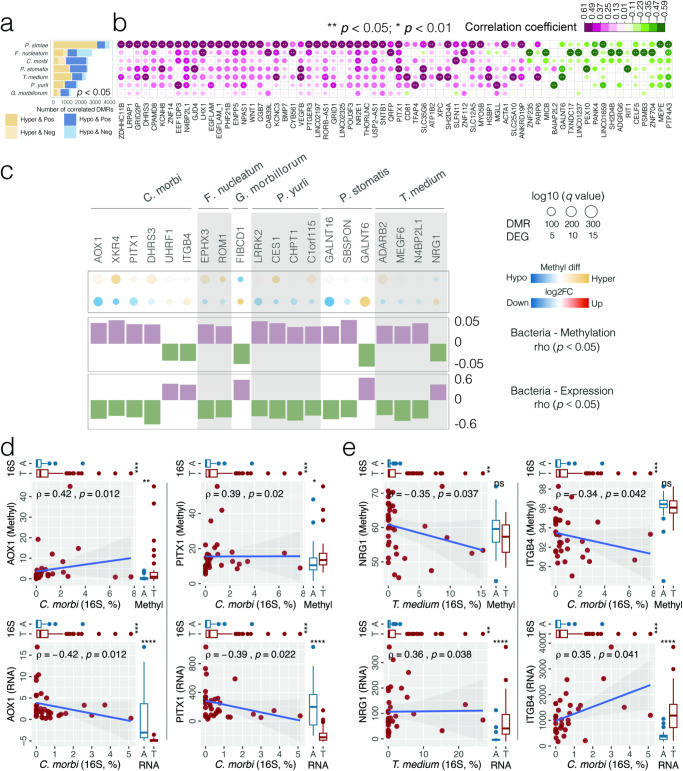
Bacteria-associated host gene epigenetic aberrance dysregulating transcriptome expression in OSCC. **a** Bar plot of the bacteria-methylation Spearman correlation showing the abundance of host DMRs in OSCC tumor tissues associated with tumor-enriched bacterial species (*p* < 0.05). Hyper and Hypo indicate hyper-methylated and hypo-methylated promoter regions, respectively; Pos and Neg indicate positive and negative association, respectively. **b** Heat map depicting highly significant bacteria-methylation correlations (*p* < 0.01). Asterisks indicate significance level (**p < 0.01; *p < 0.05). Columns are ranked by the average correlation coefficients of DMRs with tumor-enriched bacteria. **c** Host genes with opposite methylation and expression patterns that are both associated with the enrichment of tumor-enriched bacterial species in OSCC. Status of CpG methylation (DMR) and transcriptome expression (DEG) are represented by filled circles, with sizes corresponding to the *q* values. The bar plot shows Spearman correlation coefficients of CpG methylation and transcriptome expression with the abundance of tumor-enriched bacteria. **d** Scatter plots depicting *AOX1* and *PITX1* hypermethylation (top panel) and down-regulated expression (bottom panel) in relation to *C. morbi*, where the strength of correlation (Spearman rho) and significance (*p* value) are shown in each plot. Marginal box*p*lots depict overall gene expression/methylation (right) and bacterial 16S rRNA gene abundance (top), with Mann–Whitney *U* test between tumor and AN tissues. **e** Scatter plots depicting *NRG1* and *ITGB4* hypomethylation (top panel) and up-regulated expression (bottom panel) in relation to *T. medium* and *C. morbi*, respectively.

Enrichment of pathogenic bacteria in OSCC tumor microenvironment may lead to dysregulation of epigenetic modifications. To test this hypothesis, we established the association between DMRs and tumor-enriched bacterial species using Spearman correlation (Supplementary Table 15a). Overall, 13,172 DMRs (responsible for 8690 genes) and 7 tumor-enriched bacterial species formed 16,630 bacteria-methylation associated pairs (*p* < 0.05), with *P. simiae* and *F. nucleatum* contributing to 41.7% (3020/7234) of positive associations with hypermethylation (Hyper & Pos) and 50.4% (1071/2125) of negative associations with hypomethylation (Hypo & Neg), respectively (Fig. 4a, b). An integrative analysis of bacteria-methylation and bacteria-transcriptome correlations further identified 15 suppressed DEGs that might be silenced by bacteria-associated CpG hypermethylation in their promoter regions (Fig. 4c and Supplementary Table 15b). For example, the enrichment of *C. morbi* was simultaneously correlated with the hypermethylation (Hyper & Pos) and inhibited gene expression (Down & Neg) of *AOX1* and *PITX1* (Fig. 4d). Other examples of tumor suppressors include *DHRS3* and *CES1*. Meanwhile, inverse correlations of *NRG1* and *ITGB4* between DNA CpG hypomethylation and gene overexpression were found to be linked to *T. medium* and *C. morbi* abundance, respectively (Fig. 4e), implying the potential of tumor-enriched bacteria to alter DNA methylation that might translate into activated gene expression. *NRG1* as an oncogene binds to and activates members of the ErbB family of receptor tyrosine kinases, triggering downstream signaling pathways, such as the *PI3K/AKT* and *MAPK/ERK* pathways, which are involved in cell growth, survival, and proliferation. *ITGB4* is aberrantly expressed in several cancers including breast, colorectal, and lung cancers. The bacteria-methylation correlations at the genus level were established and shown in Supplementary Table 16a, b.

### OSCC-related carcinogenic potential of *Fusobacterium nucleatum* in situ

**Fig. 5:**
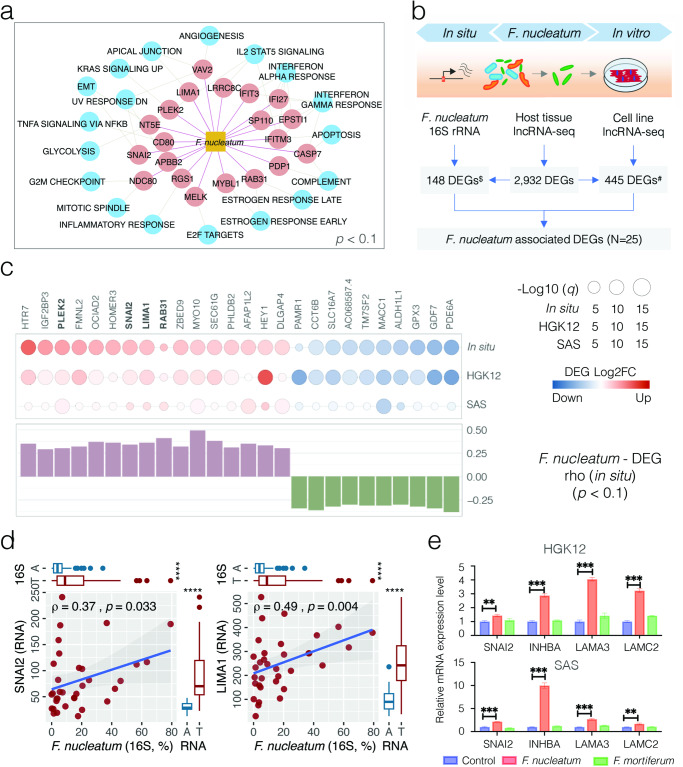
OSCC-related carcinogenic potential of *Fusobacterium nucleatum* in situ and in vitro. **a** A Gene Set Enrichment Analysis (GSEA) inferred from 384 up-regulated DEGs positively associated with 7 tumor-enriched bacterial species (Up & Pos, *p* < 0.1). The network displays 20 DEGs involving 18 hallmark pathways associated withs *F. nucleatum*. **b** Schematic illustration of an in vitro cell model by co-culturing *F. nucleatum* in human oral epithelial cells HGK12 and SAS to profile the cellular gene transcriptome using lncRNA-seq. ^$^The number of DEGs in situ (abs(log2FC > 1, *q* < 0.01) with coherent association with *F. nucleatum* 16S reads (Up & Pos and Down & Neg, *p* < 0.1); ^#^the number of DEGs in situ with coherent dysregulation in at least one cell line (abs(log2FC > 0.1, *q* < 0.01) and confirmed by another cell line (abs(log2FC > 0.1, *p* < 0.05). **c** *F. nucleatum*-associated DEGs observed simultaneously in situ and in vitro. Filled cir**c**le indicates status of transcriptome expression (DEG) in vitro and in situ, respectively, with sizes corresponding to the *q* values. The bar plot shows Spearman correlation coefficients between DEG transcriptome expression and *F. nucleatum* abundance in OSCC tumor tissues. Five *F. nucleatum*-associated GSEA hallmark genes are highlighted in bold. **d** Scatter plots depicting up-regulation of *SNAI2* and *LIMA1* in relation to *F. nucleatum* in OSCC tumor tissues, where the strength of correlation (Spearman *rho*) and significance (*p* value) are shown in each plot. Marginal boxplots depict overall gene expression (right) and bacterial 16S rRNA gene abundance (top), with Mann–Whitney *U* test between tumor and AN tissues. **e** RT-PCR analysis for the indicated overexpression of four GSEA hallmark genes (*SNAI2*, *INHBA*, *LAMA3* and *LAMC2*) in HGK12 and SAS cells co-cultured with *F. nucleatum* or *F. mortiferum*. Data are presented as mean ± SD and significance (***p < 0.001; **p < 0.01) are determined by two-tailed unpaired Student’s *t* test based on 2^−ΔΔCt^.

To better understand the potential of *F. nucleatum* in OSCC pathogenesis by altering host gene regulation, we first applied 384 up-regulated DEGs positively associated with 7 tumor-enriched bacterial species (Up & Pos, *p* < 0.1) for a Gene Set Enrichment Analysis (GSEA) (Supplementary Table 17a). Altogether 368 connections were observed (Supplementary Fig. 5 and Supplementary Table 17b), with *F. nucleatum* interacting with 20 up-regulated DEGs that involved 18 hallmark pathways (Fig. 5a). Among them, for example, *SNAI2* (log2FC = 1.90, *q* = 2.19E−11), potentially triggered by *F. nucleatum* and *C. morbi*, is one of the key transcription factors that regulates the Epithelial–Mesenchymal Transition (EMT) process. Cells undergoing EMT exhibit a loss of epithelial markers such as E-cadherin and claudins, thereby detach from the primary tumor, invade surrounding tissues, and eventually metastasize to distant sites. As *F. nucleatum* and *F. periodonticum*, the two species with close phylogenetic relationship and similar biological functions, accounted for 74.02% and 25.97% of *Fusobacterium* 16S rRNA reads, we also performed a GSEA inferred from the genus-level bacterial-transcriptome connections and observed 40 up-regulated DEGs involving 22 enriched hallmark carcinogenic pathways that were positively associated with *Fusobacterium* (Supplementary Table 17c, d), including six hallmark genes (*LAMA3*, *INHBA*, *SNAI2*, *NT5E*, *MYLK* and *TPM1*) within the EMT process (Supplementary Fig. 6).

### OSCC-related carcinogenic potential of *Fusobacterium nucleatum* in vitro

We then applied an in vitro cell-based model by co-culturing *F. nucleatum* with cancer (SAS) and non-cancer (HGK12) human oral epithelial cells, respectively, to profile cellular gene transcriptomes using lncRNA-seq (Fig. 5b and Supplementary Table 18). These two cell lines showed differentially expressed transcriptomes, with 820 up- and 478 down-regulated DEGs commonly detected (abs(log2FC) > 0.1, *q* < 0.01) (Supplementary Fig. 7a and Supplementary Table 19). While activated genes in HGK12 were involved in neutrophil mediated immune response, cytokine-mediated signaling pathway and regulation of autophagy, the gene expression in SAS infected with *F. nucleatum* was mainly enriched in the regulation of signal transduction, programmed cell death and apoptotic process (Supplementary Fig. 7b and Supplementary Tables 20 and 21), which may be in part due to that fact that these two cells have completely different properties. Interestingly, among 148 DEGs coherently associated with *F. nucleatum* 16S reads in the surveyed OSCC tumor tissues (Up & Pos and Down & Neg, *p* < 0.1), at least 15 and 10 genes were confirmed to be overexpressed or suppressed in the two co-cultured cell lines, respectively (abs(log2FC) > 0.1, *p* < 0.05) (Fig. 5b, c and Supplementary Table 19). These include the overexpression of the hallmark genes *SNAI2*, *LIMA1*, *PLEK2* and *RAB31* involved in multiple GSEA pathways (Fig. 5c, d). In contrast, *SLC16A7*, a gene encoding a membrane transport solute carrier (family 16 member 7) and commonly reduced in various types of cancer, was negatively associated with *F. nucleatum* (rho = −0.32, *p* = 0.066) and significantly suppressed in situ (log2FC = −1.71, *q* = 3.06E−05) and in vitro (HGK12: log2FC = −1.06, *q* = 1.99E−16; SAS: log2FC = −0.40, *p* = 0.008). Using a real-time PCR, we verified the overexpression of 5 representative hallmark genes involved in the EMT pathway in vitro, including *SNAI2* (*F. nucleatum*-associated), *INHBA* and *LAMA3* (*Fusobacterium*-associated), and *LAMC2* (Fig. 5e and Supplementary Table 23), although their protein levels, as measured by western blot, were controversial (Supplementary Fig. 7c and Supplementary Table 24). Our findings in vitro indicate a significant increase in the mRNA expression levels of three EMT hallmark genes (*LAMA3*, *INHBA*, *SNAI2*) upon *F. nucleatum* infection. Additionally, we included *LAMC2*, the protein-coding gene for the gamma subunit of Laminin332, which is part of the same protein complex as *LAMA3*, as supplementary evidence. We also investigated the protein laminin332 and found that the protein expressions of two subunits were potentially increased by *F. nucleatum* infection in both HGK12 and SAS cell lines. This observation aligns with the results obtained from RNAseq analysis of co-cultured cell lines but the underlying mechanisms of the carcinogenetic pathways in which these genes are involved in OSCC warrants further investigation.

## Discussion

Host-microbiota maladaptation has been reported in various types of cancer^3,16–19^. Our study through integrative analysis of bacteria-transcriptome and bacteria-methylation correlations in HPV-negative OSCC patients revealed complex networks between oral microbiota dysbiosis and host genetic and epigenetic abnormalities, functionally implicated in various cancer-related pathways. Our data also suggests that dysregulation of host gene transcriptome might be influenced by tumor-enriched bacteria or by bacteria-associated epigenetic modifications. An in vitro model further confirmed that *F. nucleatum*, an oral bacterial species closely associated with OSCC tumorigenesis, could activate hallmark genes involved in multiple cancer-associated pathways. These findings may serve as a precursor for hypothesis-driven study to better understand the molecular mechanisms of pathogenic bacteria underlying OSCC pathogenesis.

**Fig. 6:**
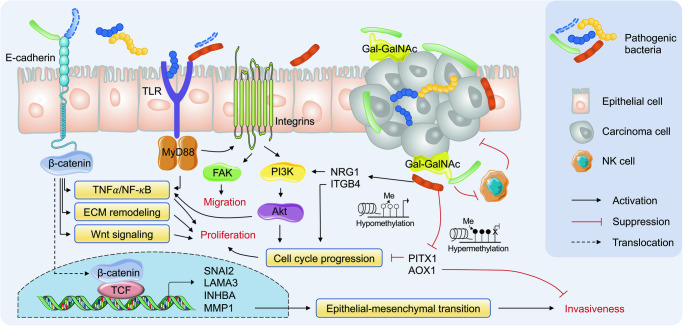
Schematic model hypothesizing the carcinogenetic potential of pathogenic bacteria in OSCC pathogenesis. *F. nucleatum* virulence factor FadA has been reported to activate the E-cadherin/β-catenin associated cell signaling, which further activates the TNF*α*/NF-*κ*B proinflammatory pathway, ECM remodeling, and Wnt signaling. Subsequently, β-catenin can be translocated to the nucleus to form a complex with T-cell factor protein (TCF) and activate EMT-related genes, such as *SNAI2*, *LAMA3*, *INHBA*, and *MMP1*. Moreover, *F. nucleatum* and *T. denticola* have been reported to promote cancer aggressivity via crosstalk between the integrin/FAK and TLR/MyD88 signaling pathways. Integrin-associated PI3K/Akt signaling can be activated by *P. anaerobius*, which regulates cell cycle progression. Gal-GalNAc on tumor cells is the receptor of Fap2 to recruit *F. nucleatum* to the tumor site. In addition, hypomethylation of *NRG1* may activate the PI3K/AKT pathway while hypermethylation of *AOX1* may promote cellular invasion and metastasis.

Carcinogenesis might arise from changes in chronic host-microbe interactions upon colonization by key pathogens. Our multi-omics data analysis highlighted several cancer-associated pathways that might be directly or indirectly triggered by tumor-enriched oral bacteria in the OSCC tumor microenvironment. For example, our integrative analysis in situ and in vitro both implied the regulatory role of *F. nucleatum* on *SNAI2* in OSCC, in line with the role of *F. nucleatum* virulence factor FadA to modulate the E-cadherin/β-catenin signaling via activating TNF*α*/NF-*κ*B proinflammatory pathway, ECM remodeling, and Wnt signaling (Fig. 6)^20^. Laminin is the main component of the extracellular matrix (ECM) and plays key functions in cell adhesion, cell migration and signal transduction^21^, among which Laminin-332 is a primary member of the laminin family and contains three chains encoded by the *LAMA3*, *LAMB3 and LAMC2*. Meanwhile, β-catenin could be translocated to the nucleus to form a complex with T-cell factor protein (TCF) and activate EMT-related genes, such as *INHBA*, *LAMA3*, *LAMC2*, *NT5E* and *MMP1*, thereby contributing to the inflammatory and oncogenic responses. Cells undergoing EMT exhibit loss of epithelial markers such as occludins, claudins, and E-cadherin, and acquire concomitant expression of Vimentin, N-cadherin, and Fibronectin^22^, in which SLUG protein (SNAI2) has a fundamental role by suppressing several cell-cell adhesion genes including E-cadherin expression^23^. Additionally, the pro-inflammatory cytokine *TNFα* acts as an inflammatory mediator to trigger EMT of tumor cells and promote metastasis^24^. It could also induce SNAI family stabilization through activation of the NF-κB pathway^25,26^.

In addition to *F. nucleatum*, several other oral bacteria, such as *Treponema denticola*, could promote cancer aggressivity via crosstalk between the integrin/FAK and TLR/MyD88 signaling pathways^7,27,28^. Integrin-associated PI3K/Akt signaling has been reported to be activated by *Peptostreptococcus anaerobius*, which regulates cell cycle progression^29^. Both bacterial species were found to be enriched in the surveyed OSCC tumor tissues with limited statistical significance. Interestingly, *T. medium* and *P. stomatis*, two bacterial species phylogenetically related to *T. denticola* and *P. anaerobius*, respectively, significantly increased in relative abundance in the OSCC tumor microenvironment. *P. stomatis* has been associated with various oral diseases even cancer^30^. For example, *Wnt7A* associated with *P. stomatis* could activate the canonical Wnt pathway through β-catenin/MMP9-mediated signaling that regulates cell proliferation, differentiation and EMT^31,32^. Three hallmark genes related to EMT, including *GJA1*, *MMP1*, and *SNAI2*, which are capable to promote cancer cell migration and invasion, were also positively associated with *P. stomatis* in the surveyed OSCC tumor tissues^33^. It is important to note that the dysregulation of host gene expression might be influenced by the contributions from several pathogenic bacteria. *LIMA1* (belonging to apical junctions) initially identified as a differentially expressed gene in oral epithelial cell carcinogenesis^34^, for example, were positively associated with *C. morbi*, *F. nucleatum* and *P. stomatis*, highlighting the complexity of the host-microbiota interactions in the OSCC tumor microenvironment. The effects of *LIMA1* can extend from cell migration and cytoskeleton dynamics to cell cycle, gene regulation, angiogenesis, and lipid metabolism, providing new ideas for future exploration of cancer treatment strategies targeting this gene^35^.

Recent studies suggest that epigenetic alterations may play important roles in the initiation and propagation of cancer^36^. Epigenetic mechanisms have also been recognized as a critical player at the interface between the human microbiome and the intestinal epithelial cell^37,38^. For example, exposure to commensal microbiota induced localized DNA methylation changes, which is necessary for proper intestinal homeostasis^39^. In contrast, *F. nucleatum* and *H. hathewayi*, two bacterial pathogens associated with colorectal cancer, are able to drive host colonic epithelial cell promoter hypermethylation of tumor suppressor genes^12^. Our data suggest that dysregulation of host gene transcriptome could be influenced by bacteria-associated epigenetic abnormality. For example, hypermethylation and downregulation of *PITX1* and *AOX1* were both associated with *C. morbi*. *PITX1* has been identified as a potential tumor-suppressor gene related to cell apoptosis and was down-regulated by DNA hypermethylation in gastric cancer^40^ and esophageal squamous cell carcinoma (ESCC)^41^. *PITX1* suppresses tumorigenicity by downregulating the RAS pathway through RASAL1, a RAS-GTPase-activating protein^42^. Similarly, loss of *AOX1*, probably linked to its hypermethylated promoter, contributes to the transition from low-grade to high-grade during bladder carcinoma progression, thereby promoting invasion and metastasis^43^. Meanwhile, hypomethylation of several oncogenes such as *NRG1*, *ITGB4* and *GALNT6* associated with tumor-enriched bacteria suggests the potential of microbiota to promote host gene expression through epigenetic activation. For example, *GALNT6* has been shown to promote tumorigenesis and metastasis by catalyzing mucin-type O-glycosylation-mediated stabilization of MUCl and fibronectin (FN) in breast cancer cells^44^. Besides, recent study has reported that *F. nucleatum* could induce the significant decline of m^6^A modifications in CRC resulting in the improvement on CRC aggressiveness^45^. The metastasis of colorectal cancer has also been demonstrated to be promoted by *F. nucleatum* through miR-1322/CCL20 axis and M2 polarization, which indicating the importance of host-microbiome interactions^46^. However, the mechanisms by which oral microbiota programmed gene promoter methylation leads to dysregulated expression in OSCC remains to be elucidated.

Our study has several limitations. First, oral cavity biopsy from healthy participants were not included. Although AN tissues from OSCC patients well serve as controls, the joint effects of environmental exposures and clinical variables between cancer patients and healthy individuals are challenging to account for long-term confounding. Also, some examination results related to oral condition were not available, which may overlook the synergistic effect of some confounders on oral microbial dysbiosis and need to be remedied in subsequent studies. Second, prognostic evaluation for OSCC is based on clinical TNM classification, but this staging system is not sufficient for optimal prognostication and may be supplemented by other methods such as histological grading. Third, this study lacks a direct link between host gene transcriptome and DNA CpG methylation since only ten overlapping tumors were available, which warrants a more comprehensive insight into the epigenetic programming inferred from a larger cohort. However, it is interesting to observe coherent correlations through integrative analysis of bacteria-transcriptome and bacteria-methylation correlations. Especially, the dysregulation of several *F. nucleatum*-associated hallmark genes involving EMT pathways could be well verified in vitro using lncRNAseq, RT-PCR and western blot. Fourth, our study reveals association but not causality of the oral microbiota in the pathogenies of OSCC; further studies using in vivo and in vitro models will be helpful to identify specific microbiota-host connections underlying the mechanism. Last but not least, the resolution in defining bacterial species inferred from 16S rRNA V3–V4 region reads may be limited and bacterial strains cannot be taken into account in the current study. However, we provide the most representative ASV sequences and encourage further analysis based on different levels of taxonomic classification as well as traditional bacterial culture.

In summary, we applied multi-omics approaches to reveal complex networks between oral mucosal microbiota and host gene dysregulation in the pathogenesis of OSCC. We found differentially abundant oral bacteria, dysregulated host gene expression, and aberrant DNA CpG methylation in tumor microenvironment, with enriched functions related to various cancer-related pathways. Integrative analysis between bacteria-transcriptome and bacteria-methylation correlations reveals that dysregulation of host gene transcriptome might be influenced by tumor-enriched bacteria or by bacteria-associated epigenetic abnormality. Our findings extend the current understanding of the host-microbiota interactions in the pathogenesis of OSCC, which provides important insights into the genetic and functional basis as potential diagnostic markers and therapeutic targets for formulating more effective prevention and intervention strategies to manage this morbid entity.

## Methods

### Patient recruitment

A total of 98 primary OSCC patients of Han Chinese ethnicity administrated to two hospitals in Hong Kong (Prince of Wales Hospital and United Christian Hospital) were recruited between October 2015 and January 2020. Demographic and clinical information at enrollment, including age, gender, smoking, alcohol consumption, location of the tumor, T stage and N stage, were collected in Supplementary Table 2. No cases of metastasis were included in this study.

### Ethics approval

This study was approved by The Joint Chinese University of Hong Kong—New Territories East Cluster Clinical Research Ethics Committee (CREC reference numbers 2015.396 and 2017.143). All patients agreed to participate with written informed consent and were reviewed by a pathologist.

### Tissue specimen collection and DNA/RNA extraction

Tumor and AN tissues (≥5 cm away from the margin of the tumor) were collected at the time of surgery as per our previous methodology^6^. These specimens were briefly irrigated with sterile saline to wash away surface contamination and then stored at −80 °C until further use. Around 10–20 mg of fresh frozen tissues were manually homogenized into small pieces and treated with 20 µl proteinase K at 55 °C overnight. DNA and RNA from the disaggregated samples were simultaneously extracted using AllPrep DNA/RNA Mini Kit (Qiagen, Valencia, CA, USA) and eluted into 50 µl elution buffer separately following the manufacturer’s protocol. DNA from tumor tissues were tested for HPV infection using a PCR-based amplicon sequencing assay as previously described^47^.

### Microbiota 16S rRNA gene V3–V4 amplicon sequencing

Extracted DNA was used for oral microbiota profiling by sequencing the bacterial 16S rRNA gene hypervariable V3–V4 region (341F: 5′–CCT ACG GGN GGC WGC AG–3′, 806R: 5′–GGA CTA CNV GGG TWT CTA AT–3′)^6,48^. A pair of dual unique 12 bp barcodes was indexed to each amplicon set through the forward and reverse primers; successful amplicons were equally pooled and sequenced on an Illumina MiSeq using paired-end 300 bp reads. For quality control, each sequencing batch included a mock DNA community, negative controls and technical replicates.

### Microbiota 16S sequence data bioinformatics and statistical analysis

Demultiplexed short 16S reads passing quality filtering were imported into QIIME2 (v2021.4) to generate an amplicon sequence variant (ASV) table as previously described^6^. The representative reads were further assigned at the species level using pplacer by inserting into a phylogenetic tree inferred from complete sequences of the bacterial 16S rRNA gene to maximize phylogenetic likelihood^49,50^. ASVs with a total count >10 after removing reads assigned to archaea, mitochondria or chloroplasts were retained, with operational taxonomic unit (OTU) count tables showing the bacterial reads per sample at different taxonomic levels. Paired tumor and AN tissues with more than 2000 reads were retained for further analysis. A phylogenetic tree was generated by inserting the representative ASV reads into the SLIVA 128 reference database using the SATe-enabled phylogenetic placement (SEPP) method^51^. Alpha diversity of the observed bacterial ASV reads based on richness, Shannon and Simpson indexes were calculated using *diversity* in the Vegan R package. GUniFrac and Bray–Curtis distance metrics were computed inferred from the ASV profile to differentiate community compositions (beta diversity) between cancer and control groups using permutational multivariate analysis of variance (PERMANOVA) with 9999 permutations using the *adonis2* in the Vegan R package^52^. In the effect size analysis, disease status (tumor vs. AN) controlled association between clinical variables, including gender, age, smoking, alcohol consumption, N and T stages, were performed. Discriminative bacterial taxa between tumor and AN tissues based on OTU count tables were estimated using linear discriminant analysis (LDA) effect size (LEfSe) analysis^53^, with a cutoff of LDA > 3 (*q* < 0.05), which was further validated by different compositional aware tools with bias correction, including ANCOM-BC2^54^, ALDEx2^55^ and ZicoSeq^56^, adjusted for the covariates of T stage and smoking (*q* < 0.05). In addition, a linear regression analysis test, non-parametric Mann–Whitney Wilcoxon rank sum test (MWU), Tukey’s honest significant difference (Tukey HSD) post hoc test, and a binomial generalized log-linear model in EdgeR^57^ were applied. Heatmaps of the most discriminative bacterial genera were generated using the *heatmap.2* in the gplots R package, with hierarchical clustering setting of “*hclust(method* = *“ward.D”)* and *dist(method* = *“euclidean”)*”. Logistic regression and receiver operating characteristic (ROC) curve with the calculation of area under the ROC curve (AUC) were used to assess the potential biomarkers identified for cancer case screening. Kaplan–Meier analysis was used for univariate survival analysis with log-rank test to determine statistical differences in survival outcomes. An optimal cutoff point inferred from the relative abundance of individual bacterial taxa in the surveyed samples was calculated to divide tumor tissues into “high” and ‘low” groups using the *cutpointr* R script. The Cox proportional hazard regression method in stepwise manner controlling for gender, age, post-surgery treatment and other factors reported significant in the univariate analysis was used for multivariate analysis of survival. A two-sided *p* value < 0.05 and/or a false discovery rate (FDR)-adjusted *p* value (*p*_adj_ or *q*) < 0.05 was used as the threshold for significance.

### HPV detection and genotyping

HPV genotyping was performed using a PCR-based amplicon sequencing assay targeting the conserved L1 open reading frame (ORF) of HPV as previously described^47^. In brief, a pair of dual 12-bp barcodes was indexed to the PCR amplicon using forward and reverse primers for demultiplexing. Short reads generated by Illumina MiSeq PE150 were blasted against a comprehensive PV reference database using UPARSE^58^. An operational taxonomic unit (OTU) count table was created using a 90% identity threshold, assigning each OTU with a PV type^59^. Based on the IARC classification, 12 HPV types (HPV16, 18, 31, 33, 35, 39, 45, 51, 52, 56, 58, 59) were ranked as high risk (HR) and considered oncogenic^60^.

### Host gene long non-coding RNA sequencing (lncRNA-seq) and transcriptome profiling

Tumor and AN tissues from a subset of 40 randomly selected OSCC patients were profiled for host genome-wide transcriptome using lncRNA-seq. In brief, total RNA was depleted for rRNA using QIAseq FastSelect rRNA remove kit and converted to directional RNA library using NEBNext Ultra II Directional RNA Library Prep Kit for Illumina NovaSeq using paired-end 150 bp reads. Pair-end sequencing reads were mapped to the hg38 human reference genome using STAR^61^. Number of reads mapped to the gene exon regions was counted using *featureCounts*^62^; genes with a mean Transcripts Per Kilobase Million (TPM) greater than 1 were retained. Differentially expressed genes (DEGs) between tumor and AN tissues were identified using a binomial generalized log-linear model in EdgeR^57^ using a cutoff of *q* < 0.01 and log2FC (fold change) > 1 (up-regulated genes) or <−1 (down-regulated genes). Age, smoking, alcohol consumption, and gender were included as confounding factors in the model. Functional enrichment of OSCC-associated DEGs were summarized using ToppGene Suite^63^. The top 10 Kyoto Encyclopedia of Genes and Genomes (KEGG) terms with statistical significance (*p* < 0.05) were visualized in order. To determine DEGs associated with disease-specific survival, the expression of each gene was labeled with a binary value of “High” or “Low” using “optimal” cutpoints determined by *cutpointr* function in R. The parameter of “metric” in cutpointr was set as “sum_sens_spec” to maximize the sum of sensitivity and specificity. The Kaplan–Meier method with 95% CI for Cox proportional hazards ratio was used for univariate survival analysis using *coxph* function in R.

### The Cancer Genome Atlas (TCGA) OSCC RNA-seq data analysis

A TCGA OSCC RNA-seq HTSeq raw read counts matrix along with metadata information (age, smoking, alcohol consumption, gender, and site of resection or biopsy) were downloaded using the TCGAbiolinks R package^64^. This dataset contains tissue samples from 502 OSCC patients and 44 non-OSCC controls. Samples were further filtered using the following criteria: (1) one sample was removed due to the lack of “age” information, and (2) 206 samples were discarded because the “site of resection or biopsy” didn’t belong to oral cavity, resulting in 309 cases and 30 controls for the comparison with the Hong Kong (HK) OSCC cohort.

### Network analysis between bacterial abundance and host gene expression

Host DEGs and OSCC-associated bacterial species were explored for bacteria-transcriptome interactions with Spearman correlation coefficients calculated using the *cor.test* function in R. Bacterial count tables were normalized in percentage for the correlation analysis. A *p* value < 0.05 was used as the cutoff for significance. Sparse Correlations for Compositional Data (SparCC)^65^ was used to explore bacteria-bacteria correlations, with a correlation coefficient *q* < 0.1 for significance. Heatmaps and networks were plotted using *corrplots* function in R and Cytoscape v3.7.1^66^, respectively. To characterize bacteria target pathways in OSCC pathogenesis, up-regulated host DEGs with positive association with tumor-enriched bacterial species were summarized for Gene Ontology (GO) biological processes using ToppGene suite^63^. The obtained *p* values were then corrected for multiple testing using the Benjamini & Hochberg (B & H) method. Enriched GO terms (*q* < 0.001) and corresponding *q* values were used as the input for REVIGO^67^ to reduce GO term redundancy and visualize semantic clustering of the identified GO terms. Meanwhile, DEGs associated with tumor-enriched bacteria and their correlation coefficients were merged for a refined hallmark gene set enrichment analysis against the Molecular Signatures Database (MSigDB) using GSEA^68^. The hallmarks effectively summarized most of the relevant biological processes of gene sets by reducing variation and redundancy.

### Human DNA CpG methylation and bioinformatic analysis

Tumor and AN tissues from a subset of 38 randomly selected OSCC patients were characterized for host DNA CpG methylation using bisulfite sequencing. In brief, the total DNA was pre-prepared for illumina library using KAPA HTP Library Preparation Kit (Kapa Biosystems, USA). The library was exposed to sodium bisulfite using EpiTect DNA Bisulfite Kit (Qiagen, USA) and then probed with Methyl-Seq Capture probes using SeqCap Epi CpGiant Kit (Roche, USA) for Illumina NovaSeq using paired-end 150 bp reads. Paired-end sequencing reads were trimmed using Trimmomatic^69^ and aligned to the human reference genome (hg38) using Bismark^70^. BAM files were sorted and deduplicated; only genomic regions with read coverage larger than 10 were included. Methylation levels were modeled using a logistic regression-based algorithm with overdispersion correction and Chi-square test implemented in the methylKit R package^71^. Differentially methylated regions (DMRs) in tumors compared with AN tissues were identified using a cutoff of *q* < 0.01 and *meth.diff* score > 1.0 (hypermethylation) or <−1.0 (hypomethylation). Age, gender, smoking, alcohol consumption, N and T stages were included as confounding factors in the model. DMRs were then assigned to the promoter region of genes using the *annotatePeak* function in the ChIPseeker R package^72^. The promoter region was defined as 3000 bp upstream and 3000 bp downstream of the transcription start sites (TSSs). When multiple DMRs were located within the same promoter region, we used a majority voting method to determine the methylation status of the gene. Spearman’s rank-order correlation test was used to explore the association between promoter-associated DMRs and OSCC-enriched bacterial species. A *p* value < 0.05 was considered as statistical significance.

### *Fusobacterium nucleatum* co-culture with host epithelial cell in vitro

To validate the potential of pathogenic bacteria to dysregulate host transcriptome revealed in OSCC tumor microenvironment, an in vitro cell-based model was established by co-culturing *F. nucleatum* in HGK12 cell, a healthy human gingival keratinocyte immortalized head and neck derived epithelial cell line (gifts from Prof. Lui, School of Biomedical Sciences, The Chinese University of Hong Kong) and SAS, a human oral tongue squamous cell carcinoma cell line (Cellosaurus, RRID:CVCL_1675). In brief, *F. nucleatum* subsp. *vincentii*, a strain isolated from an OSCC patient and confirmed by whole genome sequencing, was applied for cell-bacteria co-culture system at a multiplicity of infection (MOI) of 100:1 for 5 h at 37 °C under anaerobic condition. HGK12 and SAS were co-cultured with *F. nucleatum* once and infected cells were then incubated with fresh Epilife or DMEM/F12 medium, respectively, at 37 °C with 5% CO_2_. After overnight of culture with or without bacteria, total RNA from cells was extracted using RNeasy Mini Kit (Qiagen, USA), followed by Illumina lncRNA-seq library preparation. Three independent repeats were performed for statistical power. In brief, pair-end sequencing reads were mapped to the hg38 human reference genome using STAR^61^. Number of reads mapped to the gene exon regions was counted using *featureCounts*^62^; genes with a mean Transcripts Per Kilobase Million (TPM) greater than 1 were retained. Differentially expressed genes (DEGs) between treated and untreated cell lines were identified using a binomial generalized log-linear model in EdgeR^57^, with a cutoff of abs(log2FC) > 0.1 and *q* < 0.01 considered statistically significant. Functional enrichment of *F. nucleatum*-associated DEGs were summarized using ToppGene Suite^63^. The top 10 Kyoto Encyclopedia of Genes and Genomes (KEGG) terms with statistical significance (*p* < 0.05) were visualized in order.

### Real-time PCR (RT-PCR) of mRNA gene expression

Total RNA was extracted from HGK12 and SAS cell lines co-cultured with *F. nucleatum* (MOI 1:100) by using TRIzol reagent. As a negative-bacterial control, *Fusobacterium mortiferum*, a suggested non-invasive and non-pathogenic species within the *Fusobacterium* genus, was utilized^73^. One µg of total RNA was applied for generating complementary DNA by LunaScript® RT SuperMix Kit (NEB, #E3010) followed by standard RT-PCR reaction with Luna® Universal qPCR Master Mix Protocol (NEB, #M3003). The RT-PCR primers used for gene expression validation are listed in Supplementary Table 22.

### Western blot of protein expression

Protein samples were obtained from co-cultured HGK12 and SAS cell lines in the presence or absence of respective bacteria. The samples were collected using RIPA lysis buffer (Santa Cruz). The protein quantification was performed using Pierce™ BCA Protein Assay Kits (Thermo Fisher Scientific). A total of 20 µg of protein from each sample was separated by 10% or 12% SDS-PAGE and transferred onto PVDF membranes for blotting. To prevent nonspecific binding, all membranes were blocked using 5% non-fat milk in 1X TBST. Primary antibodies against *SNAI2* (Cell Signaling Technology, 9585T, 1:500 dilution), *INHBA* (Abcam, ab128958, 1:500), *LAMA3* (Abcam, ab151715, 1:500), *LAMC2* (Santa Cruz, sc-28330, 1:1000) and β actin (Santa Cruz, sc-47778, 1:1000), were then incubated overnight at 4 °C, respectively, followed by the application of corresponding secondary antibodies for 1 h at room temperature. The target protein bands were detected using Clarity Western ECL Substrate (Bio-Rad) and imaged using Chemiluminescent Western Blot Imager AZURE 300 (Azure Biosystems). All expression levels were determined by quantifying the grayscale values of the target protein bands relative to the reference beta-actin using ImageJ software. All blots and gels derive from the same experiment and they were processed in parallel.

### Reporting summary

Further information on research design is available in the Nature Research Reporting Summary linked to this article.

### Supplementary information

Supplementary Figures

nr-reporting-summary

Supplementary Tables

Supplementary Data 1

Supplementary Data 2

Supplementary Data 3

## Data Availability

All sequences and codes are available upon request. The 16S rRNA gene amplicon next-generation sequences analyzed in this study are available in NCBI SRA database under BioProject PRJNA822685 (accession numbers from SRR18595688 to SRR18595849). The bioinformatics pipelines and scripts used in this study have been deposited to the Github repository (https://github.com/lycai05/OSCC_host_bacteria_interactions).
