# Supplementary material for: Integrative analysis reveals associations between oral microbiota dysbiosis and host genetic and epigenetic aberrations in oral cavity squamous cell carcinoma
Source: NPJ Biofilms Microbiomes. 2024 Apr 8;10:39. doi: 10.1038/s41522-024-00511-x (PMC11001959; doi:10.1038/s41522-024-00511-x)
Supplement: Supplementary file 1 — Supplementary Figures [file 41522_2024_511_MOESM1_ESM.pdf]

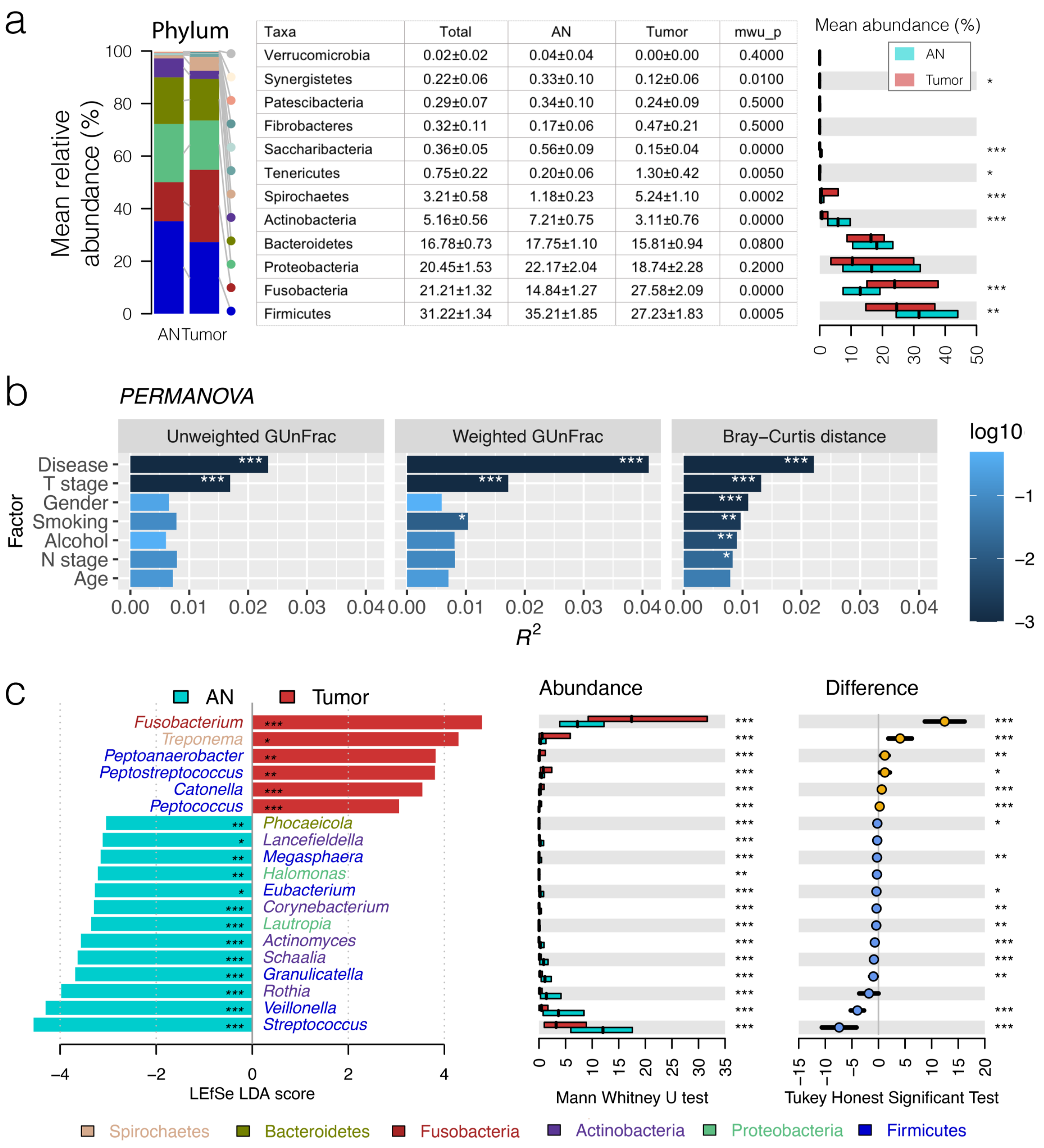

Taxa	Total	AN	Tumor	mwu_p
Verrucomicrobia	0.02±0.02	0.04±0.04	0.00±0.00	0.4000
Synergistetes	0.22±0.06	0.33±0.10	0.12±0.06	0.0100
Patescibacteria	0.29±0.07	0.34±0.10	0.24±0.09	0.5000
Fibrobacteres	0.32±0.11	0.17±0.06	0.47±0.21	0.5000
Saccharibacteria	0.36±0.05	0.56±0.09	0.15±0.04	0.0000
Tenericutes	0.75±0.22	0.20±0.06	1.30±0.42	0.0050
Spirochaetes	3.21±0.58	1.18±0.23	5.24±1.10	0.0002
Actinobacteria	5.16±0.56	7.21±0.75	3.11±0.76	0.0000
Bacteroidetes	16.78±0.73	17.75±1.10	15.81±0.94	0.0800
Proteobacteria	20.45±1.53	22.17±2.04	18.74±2.28	0.2000
Fusobacteria	21.21±1.32	14.84±1.27	27.58±2.09	0.0000
Firmicutes	31.22±1.34	35.21±1.85	27.23±1.83	0.0005

Supplementary **Fig. 1: Mucosal oral microbiota dysbiosis in patients with OSCC. a** The composition of oral microbiota summarized at phylum level between tumors and the adjacent normal (AN) tissues. Percentage of each bacterial phylum was plotted in the bar chart, with mean abundance ± standard error of the mean in the table on the right panel. **b** Effect size (R^2 value) of variables on the oral microbiota in patients with OSCC. Disease status was adjusted for covariates (gender, age, smoking, alcohol consumption, T and N stages) in *adonis2*. **c** Discriminative bacterial genus as detected by linear discriminant analysis (LDA) effect size (LEfSe) analysis (score > 3, $q < 0.05$), which was further validated by at least two of the three compositional aware tools, ANCOM-BC2, ALDEx2 and ZicoSeq tests adjusted for the covariates of T stage and smoking ($q < 0.05$). The bar length represents log10 LDA score. Differences in the relative

abundance were further tested by pairwise Mann-Whitney U test and Tukey HSD post hoc as shown on the right panels. *, $p < 0.05$; **, $p < 0.01$; ***, $p < 0.001$.

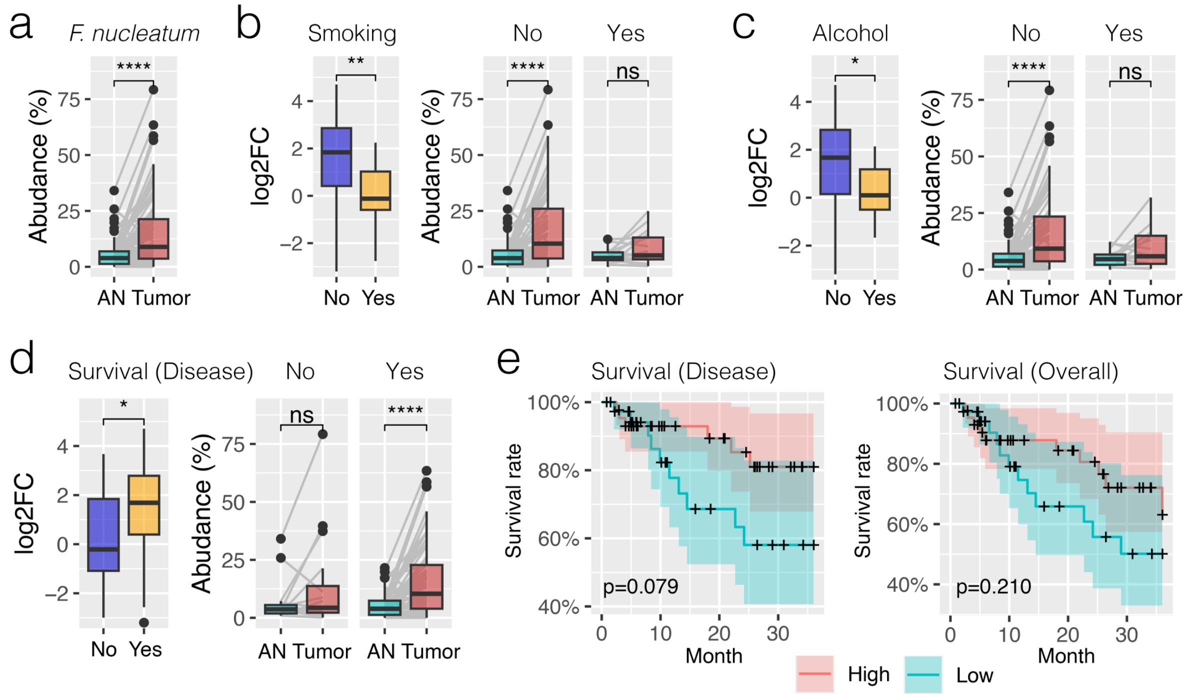

Supplementary **Fig. 2: Association of *F. nucleatum* abundance with OSCC patient outcomes.** **a** Relative abundance of *F. nucleatum* between tumors and AN tissues. High abundance of *F. nucleatum* was positively associated with **b** non-smokers, **c** non-drikers, and **d** better disease specific survival. **e** A higher relative abundance of *F. nucleatum* in tumor tissue was correlated with improved 3-year disease-specific survival. The center lines of the boxplots in **a-d** indicate the median value, the box bounds represent the first and third quartiles, and the whiskers extend to the smallest and largest values in the data, respectively.

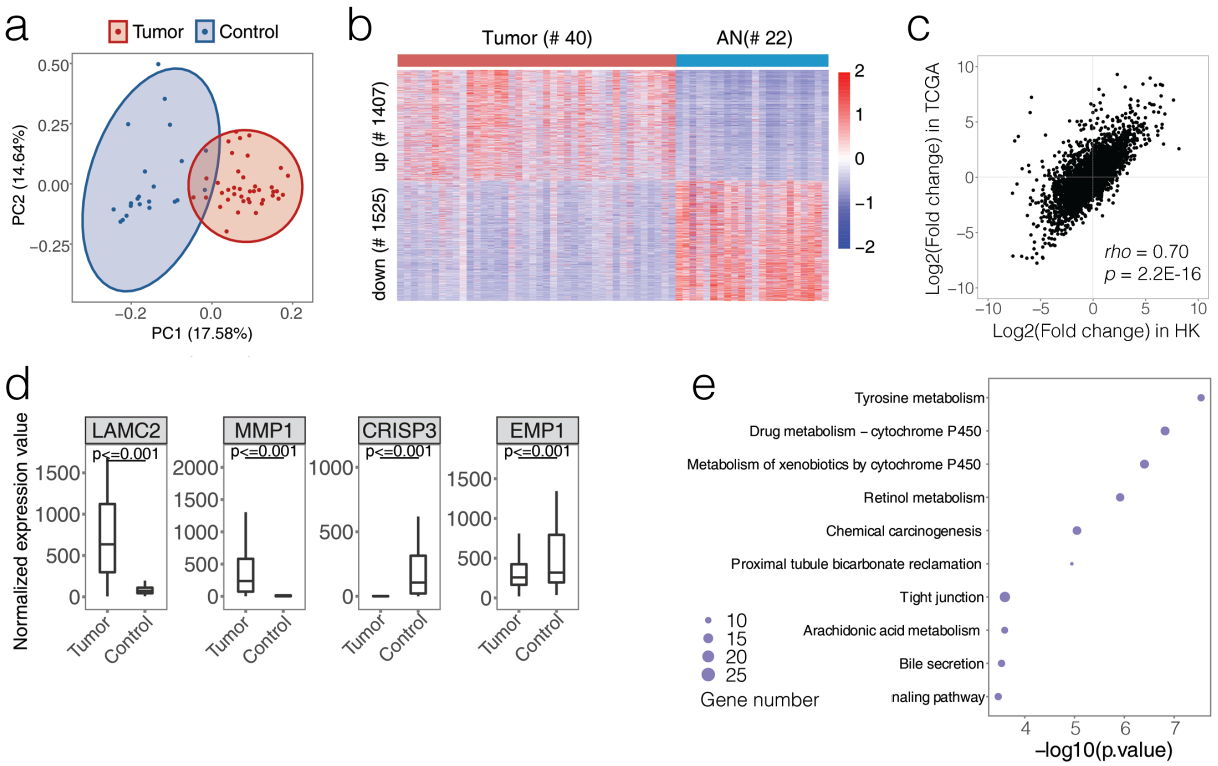

Supplementary Fig. 3: Profiling of host gene transcriptome in OSCC. a Principle coordinate analysis (PCA) distinguishing tumor (N=40) and AN (N=22) tissues inferred from the normalized expression levels of 17,225 genes with mean transcripts per kilobase million greater than 1 (TPM > 1). Dots in red and blue represent tumor and AN tissues, respectively. **b** Heat map showing up-regulated and down-regulated differentially expressed genes (DEGs) in surveyed OSCC tissues. The expression levels are normalized and scaled by z-score values. **c** Scatter plot comparing the expression levels of DEGs between HK-OSCC (40 tumors and 22 AN tissues) and TCGA-OSCC (309 tumors and 30 healthy controls) cohorts, where the strength of correlation (Spearman *rho*) and significance (*p* value) are shown in the lower right corner of the plot. **d** Box plot of four representative genes (*LAMC2*, *MMP1*, *EMP1*, *CRISP3*) showing differential expression between tumors and healthy controls in TCGA-OSCC cohort. The boxplot's center line indicates the median value, the box bounds represent the first and third quartiles, and the whiskers extend to the smallest and largest values in the data, respectively. **e** Top ten Kyoto Encyclopedia of Genes and Genomes (KEGG) pathways significantly enriched by down-regulated DEGs in OSCC ($q < 0.05$). Sizes of the circles indicate the number of hitting genes in the pathway.

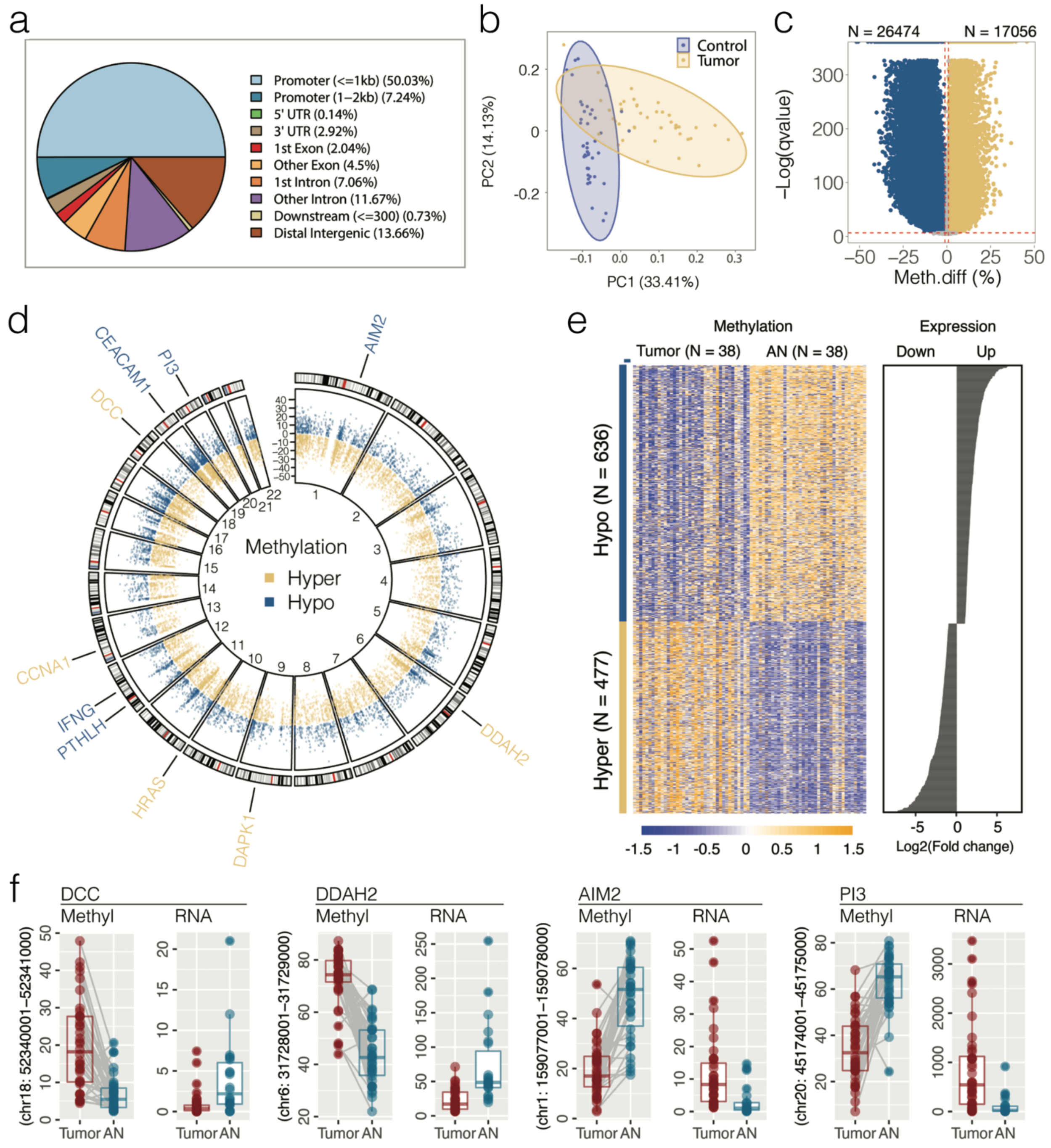

Supplementary Fig. 4: Identification of DNA CpG methylation (Methyl) correlated to host genes in OSCC. a Distribution of CpG islands with at least 10 reads per sample showing that 57.27% of sites were located within promoter regions. **b** Principle coordinate analysis (PCA) distinguishing tumors (N=38) and the paired NA tissues (N=38) inferred from 1kb window genome-wide methylation profiles. Dots in orange and blue represent tumors and AN tissues, respectively. **c** Volcano plot showing differentially methylated promoter regions in OSCC tumors when compared to the paired AN tissues. Hyper- (N=17,056) and hypo-methylated (N=26,474) differentially methylated regions (DMRs) are indicated in yellow and blue, respectively (abs(meth.diff) > 1%, $q < 0.01$). **d** The Circos plot profiling the genome-wide CpG methylation

(inner ring) between paired OSCC tumor and AN tissues. Dots in orange and blue represent genes hyper- (Hyper) and hypo- (Hypo) methylated in their promoter regions by methylKit (abs(meth.diff) > 1, $q < 0.01$). The genomic position of some previously reported methylation genes are annotated in the outer ring. **e** Heat map of differentially methylated regions (DMRs) with opposite expression levels in the HK-OSCC cohort. Methylation levels are z-score normalized, with genes sorted by log2FC expression level. **f** Box plot of representative genes (*DCC*, *DDAH2*, *AIM2*, *PI3*) with opposite methylation and transcriptome expression. The boxplot's center line indicates the median value, the box bounds represent the first and third quartiles, and the whiskers extend to the smallest and largest values in the data, respectively.

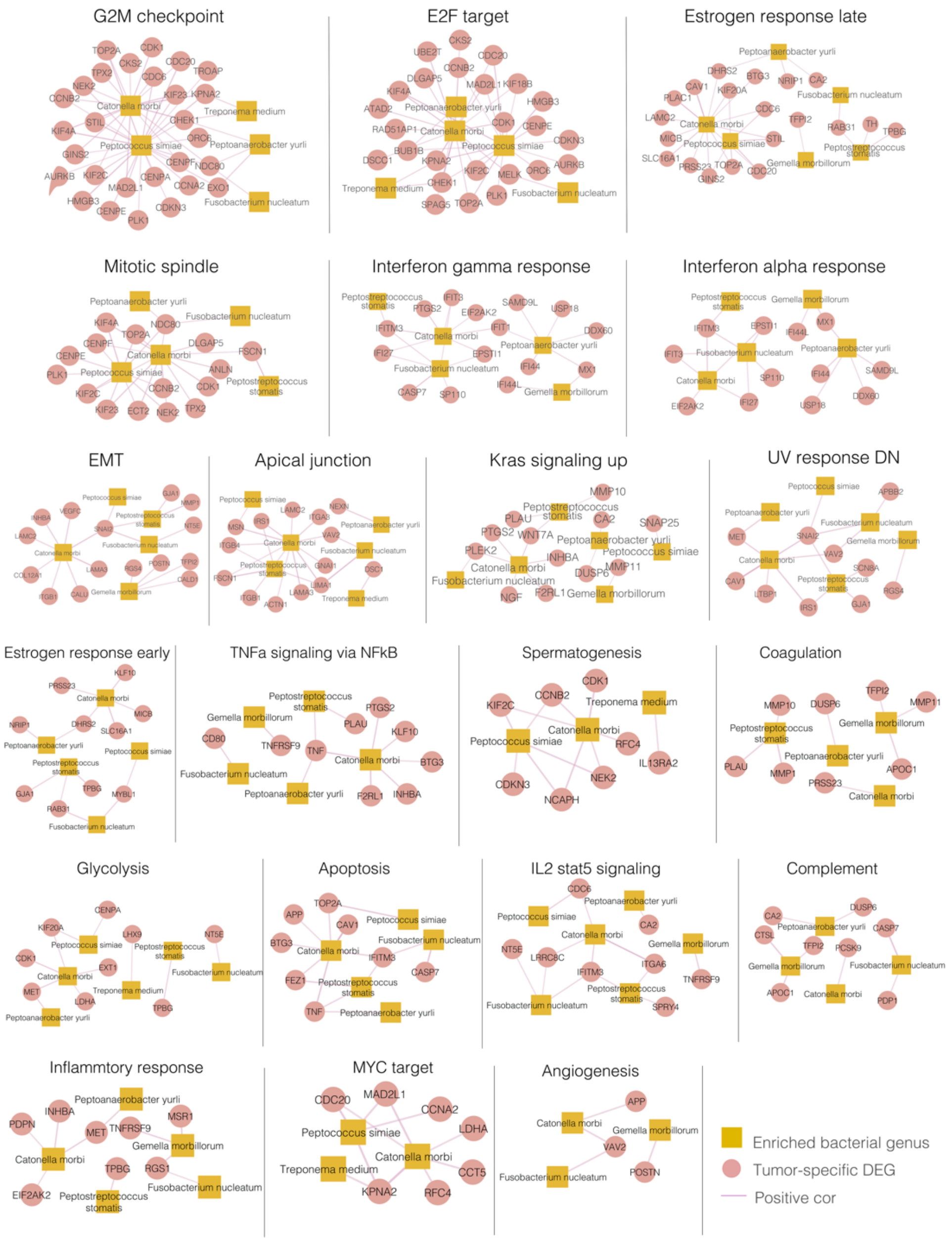

Supplementary Fig. 5: A GSEA analysis inferred from 384 up-regulated DEGs positively associated with 7 tumor-enriched bacterial species (Up & Pos, $p < 0.1$) found 130 DEGs (368 bacteria-transcriptome connections) involving 21 hallmark pathways were potentially triggered by tumor-enriched bacteria.

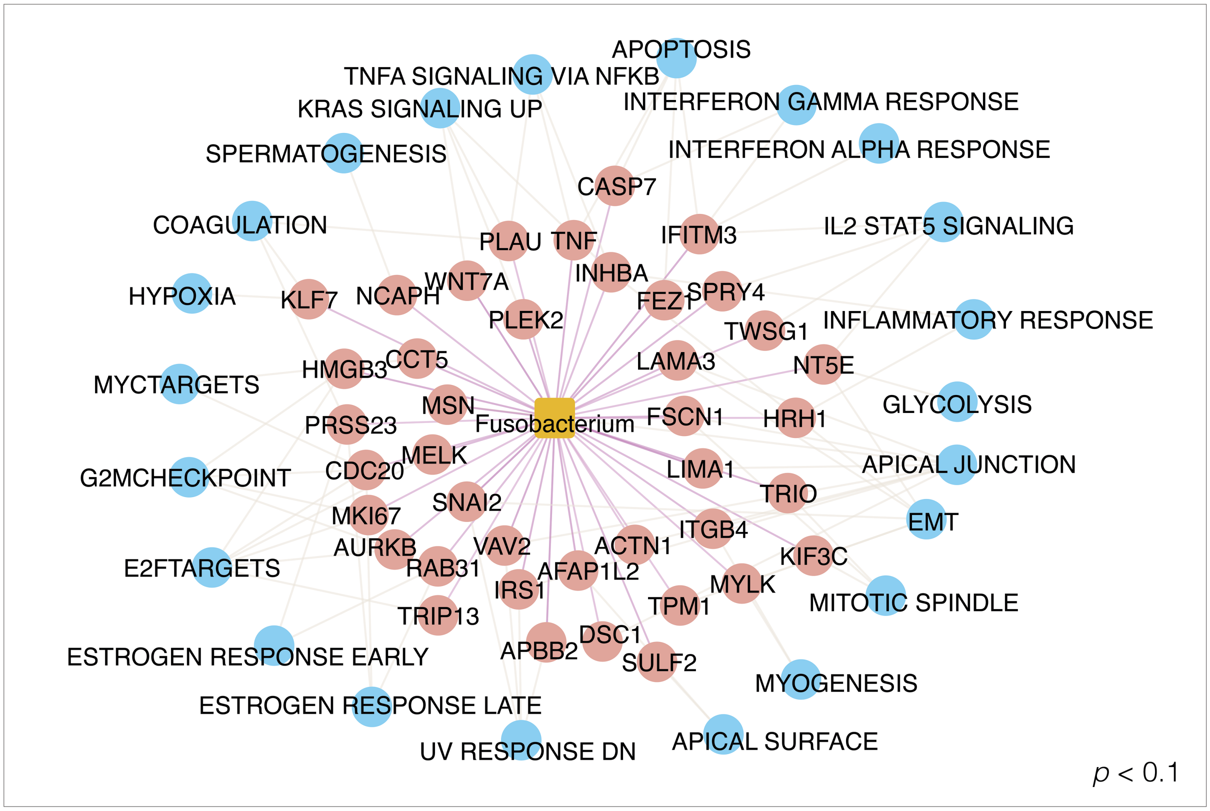

Supplementary Fig. 6: A Gene Set Enrichment Analysis (GSEA) inferred from 330 up-regulated DEGs positively associated *Fusobacterium* genus (Up & Pos, $p < 0.1$) found 40 DEGs involving 22 hallmark pathways.

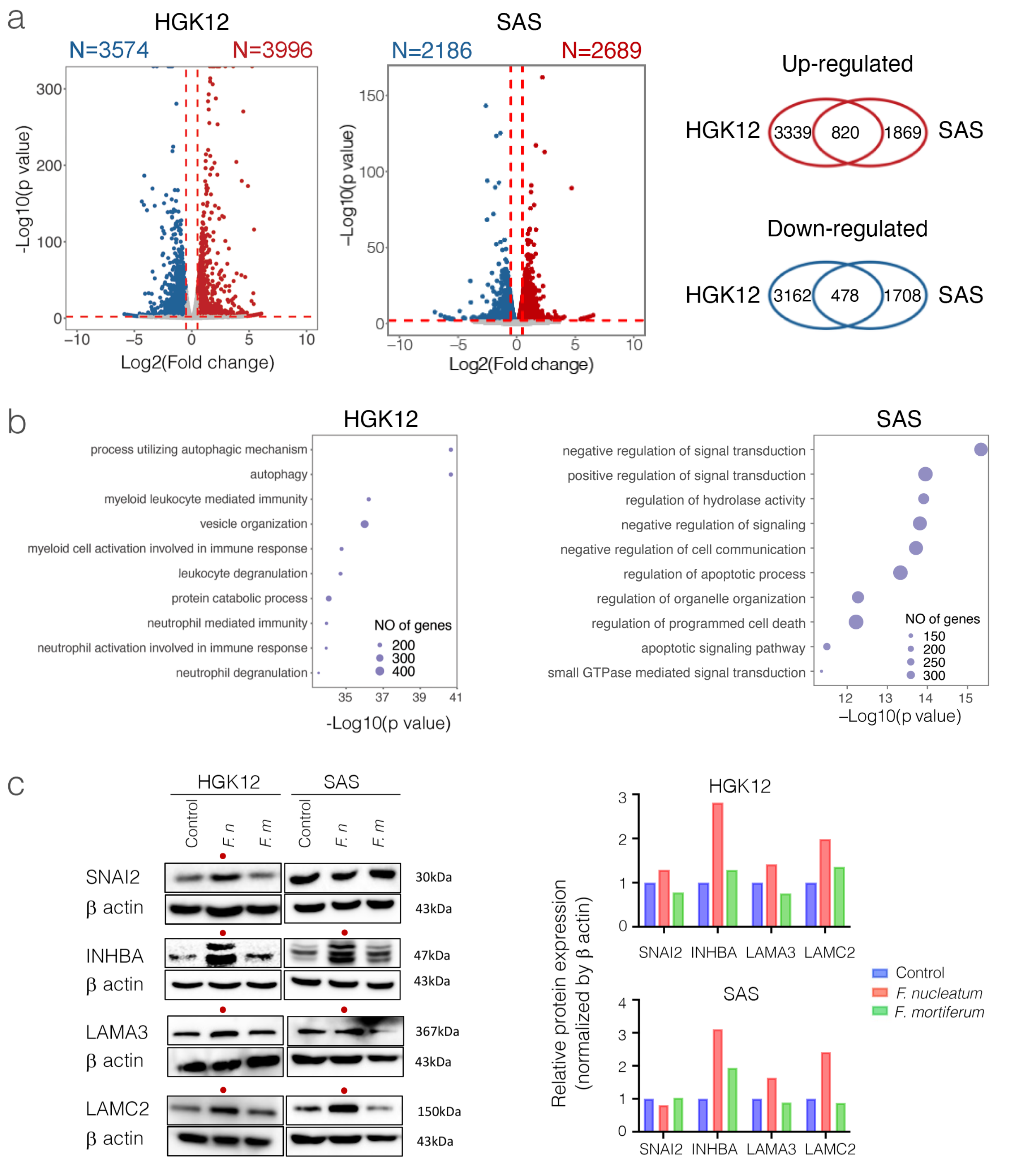

Supplementary Fig. 7: Profiling of host gene transcriptome in HGK12 and SAS co-cultured with *Fusobacterium nucleatum* by lncRNA-seq. a Volcano plot showing differentially expressed genes (DEGs) in HGK12 and SAS cells co-cultured with *F. nucleatum* when compared to uninfected control identified using binomial generalized log-linear model in EdgeR (abs(log2FC) > 0.1, $q < 0.01$). Dots in red and blue indicate up- and down-regulated DEGs. **b** Top ten Kyoto Encyclopedia of Genes and Genomes (KEGG) pathways significantly enriched by up-regulated DEGs in HGK12 and SAS cells co-cultured with *F. nucleatum* ($q < 0.05$). Sizes of the circles

indicate the number of hitting genes in the pathway. **c** Western blot showing the overexpression of proteins encoded by four GSEA hallmark genes (*SNAI2*, *INHBA*, *LAMA3* and *LAMC2*) in HGK12 and SAS cells co-cultured with *F. nucleatum* or *F. mortiferum*. The bar charts on the right panel of the figure show the relative levels compared to the negative control. Elevated protein expressions are indicated by red dots.
