# Supplementary material for: Integrative analysis reveals associations between oral microbiota dysbiosis and host genetic and epigenetic aberrations in oral cavity squamous cell carcinoma
Source: NPJ Biofilms Microbiomes. 2024 Apr 8;10:39. doi: 10.1038/s41522-024-00511-x (PMC11001959; doi:10.1038/s41522-024-00511-x)
Supplement: Supplementary file 2 — nr-reporting-summary [file 41522_2024_511_MOESM2_ESM.pdf]

nature portfolio

Corresponding author(s): Zigui CHEN

Last updated by author(s): Feb 13, 2024

Reporting Summary

Nature Portfolio wishes to improve the reproducibility of the work that we publish. This form provides structure for consistency and transparency in reporting. For further information on Nature Portfolio policies, see our Editorial Policies and the Editorial Policy Checklist.

Statistics

For all statistical analyses, confirm that the following items are present in the figure legend, table legend, main text, or Methods section.

n/a	Confirmed	
☐	☒	The exact sample size (*n*) for each experimental group/condition, given as a discrete number and unit of measurement
☐	☒	A statement on whether measurements were taken from distinct samples or whether the same sample was measured repeatedly
☐	☒	The statistical test(s) used AND whether they are one- or two-sided<br>*Only common tests should be described solely by name; describe more complex techniques in the Methods section.*
☐	☒	A description of all covariates tested
☒	☐	A description of any assumptions or corrections, such as tests of normality and adjustment for multiple comparisons
☐	☒	A full description of the statistical parameters including central tendency (e.g. means) or other basic estimates (e.g. regression coefficient) AND variation (e.g. standard deviation) or associated estimates of uncertainty (e.g. confidence intervals)
☐	☒	For null hypothesis testing, the test statistic (e.g. *F*, *t*, *r*) with confidence intervals, effect sizes, degrees of freedom and *P* value noted<br>*Give P values as exact values whenever suitable.*
☒	☐	For Bayesian analysis, information on the choice of priors and Markov chain Monte Carlo settings
☒	☐	For hierarchical and complex designs, identification of the appropriate level for tests and full reporting of outcomes
☒	☐	Estimates of effect sizes (e.g. Cohen's *d*, Pearson's *r*), indicating how they were calculated

Our web collection on statistics for biologists contains articles on many of the points above.

Software and code

Policy information about availability of computer code

Data collection	N/A
Data analysis	R 3.6.3

For manuscripts utilizing custom algorithms or software that are central to the research but not yet described in published literature, software must be made available to editors and reviewers. We strongly encourage code deposition in a community repository (e.g. GitHub). See the Nature Portfolio guidelines for submitting code & software for further information.

Data

Policy information about availability of data

All manuscripts must include a data availability statement. This statement should provide the following information, where applicable:

- Accession codes, unique identifiers, or web links for publicly available datasets
- A description of any restrictions on data availability
- For clinical datasets or third party data, please ensure that the statement adheres to our policy

All sequences and codes are available upon request. The 16S rRNA gene amplicon next-generation sequences analysed in this study are available in NCBI SRA database under BioProject PRJNA822685 (accession numbers from SRR18595688 to SRR18595849). The bioinformatics pipelines and scripts used in this study have been deposited to the Github repository (https://github.com/lycai05/OSCC_host_bacteria_interactions).

Research involving human participants, their data, or biological material

Policy information about studies with human participants or human data. See also policy information about sex, gender (identity/presentation), and sexual orientation and race, ethnicity and racism.

Reporting on sex and gender	This study does not address this issue
Reporting on race, ethnicity, or other socially relevant groupings	The study doesn't address this issue.
Population characteristics	A total of 98 OSCC patients who provided paired tumor and AN tissues were recruited in this study. Among them, eight patients infected with high-risk HPV types (7 with HPV16 and one with HPV18) were excluded. This retained cohort consisted of 57 males and 33 females, with a mean age of 65 yrs (sd: 12 yrs).
Recruitment	The recruitment information could be obtained from 'Patient recruitment" in "Materials and Method" section
Ethics oversight	This study was approved by The Joint Chinese University of Hong Kong – New Territories East Cluster Clinical Research Ethics Committee (CREC reference numbers 2015.396 and 2017.143). All patients agreed to participate with written informed consent and were reviewed by a pathologist.

Note that full information on the approval of the study protocol must also be provided in the manuscript.

Field-specific reporting

Please select the one below that is the best fit for your research. If you are not sure, read the appropriate sections before making your selection.

☒ Life sciences ☐ Behavioural & social sciences ☐ Ecological, evolutionary & environmental sciences

For a reference copy of the document with all sections, see nature.com/documents/nr-reporting-summary-flat.pdf

Life sciences study design

All studies must disclose on these points even when the disclosure is negative.

Sample size	Sample size was chosen based on previous experience and standards in the field, 90 OSCC patients were included for sequencing and data analysis.
Data exclusions	A total of 98 OSCC patients who provided paired tumor and AN tissues were recruited in this study. Among them, eight patients infected with high-risk HPV types (7 with HPV16 and one with HPV18) were excluded. By only HPV-negative patients, we were able to investigate the relations between bacterial taxa and host genes.
Replication	Three biological replicates were established using cell line model.
Randomization	N/A
Blinding	N/A

Reporting for specific materials, systems and methods

We require information from authors about some types of materials, experimental systems and methods used in many studies. Here, indicate whether each material, system or method listed is relevant to your study. If you are not sure if a list item applies to your research, read the appropriate section before selecting a response.

Materials & experimental systems

n/a	Involved in the study	
☐	☒	Antibodies
☐	☒	Eukaryotic cell lines
☒	☐	Palaeontology and archaeology
☒	☐	Animals and other organisms
☒	☐	Clinical data
☒	☐	Dual use research of concern
☒	☐	Plants

Methods

n/a	Involved in the study	
☒	☐	ChIP-seq
☒	☐	Flow cytometry
☒	☐	MRI-based neuroimaging

Antibodies

Antibodies used	Primary antibodies against SNAI2 (Cell Signaling Technology, 9585T, 1:500), INHBA (Abcam, ab128958, 1:500), LAMA3 (Abcam, ab151715, 1:500), LAMC2 (Santa Cruz, sc-28330, 1:1000) and beta actin (Santa Cruz, sc-47778, 1:1000); Secondary antibody: Polyclonal Swine Anti-Rabbit Immunoglobulins/HRP (Dako, P0217, 1:2000), Polyclonal Rabbit Anti-Mouse Immunoglobulins/HRP (Dako, P0260, 1:2000)
Validation	The antibodies were all commercially available and validated by respective companies.

Eukaryotic cell lines

Policy information about cell lines and Sex and Gender in Research

Cell line source(s)	SAS is a human oral tongue squamous cell carcinoma cell line (Cellosaurus, RRID:CVCL_1675) and HGK12 cell is a healthy human gingival keratinocyte immortalized head and neck derived epithelial cell line. Both cell lines are gifts from Prof. Lui, School of Biomedical Sciences, The Chinese University of Hong Kong.
Authentication	Short tandem repeat (STR) genotyping of the HGK12 and SAS cell lines
Mycoplasma contamination	All cell lines are tested negative for mycoplasma
Commonly misidentified lines (See ICLAC register)	None
